# Loss of function of VdDrs2, a P4-ATPase, impairs the toxin secretion and microsclerotia formation, and decreases the pathogenicity of *Verticillium dahliae*

**DOI:** 10.3389/fpls.2022.944364

**Published:** 2022-08-22

**Authors:** Hui Ren, Xianbi Li, Yujie Li, Mengjun Li, Jiyuan Sun, Fanlong Wang, Jianyan Zeng, Yang Chen, Lei Wang, Xingying Yan, Yanhua Fan, Dan Jin, Yan Pei

**Affiliations:** Biotechnology Research Center, Southwest University, Chongqing, China

**Keywords:** *Verticillium dahliae*, cotton, P4 ATPases, mycotoxins, pathogenicity

## Abstract

Four P4-ATPase flippase genes, *VdDrs2, VdNeo1, VdP4-4*, and *VdDnf1* were identified in *Verticillium dahliae*, one of the most devastating phytopathogenic fungi in the world. Knock out of *VdDrs2, VdNeo1*, and *VdP4-4*, or knock down of *VdDnf1* significantly decreased the pathogenicity of the mutants in cotton. Among the mutants, the greatest decrease in pathogenicity was observed in Δ*VdDrs2*. VdDrs2 was localized to plasma membrane, vacuoles, and *trans*-Golgi network (TGN). In vivo observation showed that the infection of the cotton by Δ*VdDrs2* was significantly delayed. The amount of two known Verticillium toxins, sulfacetamide, and fumonisin B1 in the fermentation broth produced by the Δ*VdDrs2* strain was significantly reduced, and the toxicity of the crude Verticillium wilt toxins to cotton cells was attenuated. In addition, the defect of VdDrs2 impaired the synthesis of melanin and the formation of microsclerotia, and decreased the sporulation of *V. dahliae*. Our data indicate a key role of P4 ATPases-associated vesicle transport in toxin secretion of disease fungi and support the importance of mycotoxins in the pathogenicity of *V. dahliae*.

## Introduction

Verticillium wilt (VW) leads to vascular disease on many annual economic crops (e.g., cotton, lettuce, potato, cauliflower, sunflower, eggplant, and strawberry) and perennial fruit trees (e.g., olive, mango, and cacao) (Bhat and Subbarao, 1999; Fradin and Thomma, 2006; Klosterman et al., 2009; Akhlaghi et al., 2020), resulting in tremendous economic losses globally. Taking cotton as an example, VW is regarded as an important limiting factor in cotton production, which is a mainstay of many countries’ economies. In China, more than 40% of the cotton suffers by VW, leading to huge economic losses annually (Wang et al., 2016; Gong et al., 2017). Cotton VW is caused by *V. dahliae* Kleb, a soil-borne pathogen. Due to the paucity of resistance traits in upland cotton (*Gossypium hirsutum* L.) and no effective fungicide available to cure the infected plants, VW control is far from successful (Pegg and Brady, 2002; Klosterman et al., 2009), and the disease is thus called as the cancer of cotton (Wang et al., 2016).

Wilting is a typical symptom of VW disease. Although still in debate, increasing evidences support the hypothesis that the wilting symptom is resulted from toxin activity, but not vessel occlusion (Fradin and Thomma, 2006). Toxins produced by *V. dahliae*, including high molecular weight protein lipopolysaccharide complex (Buchner et al., 1982; Nachmias et al., 1982; Riaan et al., 1994), phytotoxic peptides (Abraham et al., 1985), and low molecular weight metabolites (Daly and Deverall, 1984; Fradin and Thomma, 2006), have been regarded as the key virulence factors of VW disease. Detoxification of VW toxins is now emerging as a tool to control the disease (Wang et al., 2021). However, little is known about the delivery mechanism of *V. dahliae* toxins, which limits our understanding of pathogenic processes of the disease.

P4-ATPases are ATP-fueled flippases, catalyzing the translocation of phospholipids from the exoplasmic/luminal to the cytosolic leaflet to form asymmetric distribution of lipids (Baldridge et al., 2013; Roland and Graham, 2016; Takada et al., 2018), and thus play important roles in vesicle formation and membrane trafficking in the secretory and endocytic pathways (Graham and Kozlov, 2010; Paulusma and Elferink, 2010; Sebastian et al., 2012). P4 ATPases-associated vesicle transport is implicated in many important cellular processes, including biogenesis of cellular organelles, endocytosis, and secretion (Rothman and Wieland, 1996; Hara-Nishimura et al., 1998; McMahon and Gallop, 2005; De Matteis et al., 2013; van der Mark et al., 2013; Lopez-Marques et al., 2014). It has been reported that P4-ATPases were associated with virulence of phytopathogenic fungi. Loss-of-function of MgATP2, a P4-ATPase of *Magnaporthe oryzae*, disrupted the secretion of extracellular enzymes and decreased its pathogenicity to rice (Gilbert et al., 2006). Yun et al. (2020) found that disruption of FgDnfA and FgDnfD remarkably decreased the secretion of mycotoxin deoxynivalenol (DON) production and significantly impaired the pathogenicity of the fungus to wheat (Yun et al., 2020). However, the role of P4 ATPases-mediated transport in the mycotoxin delivery is far from clear, and the association between the secretion of Verticillium toxins and P4 ATPases-associated vesicle transport remains to be investigated.

In the present study, we identified four P4-ATPase genes in *V. dahliae* genome, and revealed that disruption of VdDrs2 severely impairs the pathogenicity of *V. dahliae*. We show that VdDrs2 is localized to plasma membrane, vacuoles, and *trans*-Golgi network (TGN), and the loss of function of VdDrs2 significantly decreases the toxin secretion of the fungus. Our results indicate a key role of P4-ATPases in toxin secretion of pathogenic fungi and support the importance of mycotoxins in the pathogenicity of *V. dahliae*.

## Materials and methods

### Microbial strains and culture conditions

Vd991, a defoliating strain of *V. dahliae* (kindly provided by Professor Guiliang Jian, Institute of Plant Protection, Chinese Academy of Agricultural Sciences) and mutants resulted from this study were grown on Potato Dextrose broth/agar (PDB/PDA, 254920, BD-Difco, Sparks, NV, United States), Czapek-Dox broth/agar (CZB/CZA, 233810, BD-Difco, Sparks, NV, United States). *Escherichia coli* DH5α (9057, TaKaRa, Kyoto, Japan) and *Agrobacterium tumefaciens* AGL-1 (Lab stock) were used for plasmid constructions and fungal transformations, respectively.

### Gene cloning and bioinformatic analysis

Reference sequences of genes were downloaded from the GeneBank database. Cluster analysis was conducted using MEGA version 5.05 software. Branch lengths are indicated in a circletree. The full-length *VdDrs2* sequence was amplified from *V. dahliae g*DNA and cDNA with paired primers *VdDrs2-F*/*VdDrs2-R* (Supplementary Table 1). The amplified products were sent to Qingke Biotech Co., Ltd., (Chengdu, China) for sequencing. Coding region DNA and cDNA of *VdDrs2* were compared using DNAStar-MegAlign. The transmembrane regions and hydrophobicity profile were predicted using the TM predict program, https://services.healthtech.dtu.dk/service.php?TMHMM-2.0 (2021).

### Gene deletion, gene down-regulation, and complementation

Primers used in this study are listed in Supplementary Table 1. All PCR products were generated by PrimeSTAR MAX Premix (R045, TaKaRa, Kyoto, Japan) and cloned into target vectors using Exnase MultiS (C112-01, Vazyme, Nanjing, China) or T4 DNA ligase (CV0701, Aidlab, Beijing, China). Homologous recombination was performed for the gene deletion mutant and gene down-regulation strain constructions. Plasmids pK2-*HygR*, containing Hygromycin B resistance gene and pK2-*NeoR*, containing Geneticin (G418) resistance gene, were used as backbone to construct the transformation vectors (Supplementary Figure 1), respectively. The two resistant genes are driven by the *Aspergillus nidulans trpC* promoter and terminated by *trpC* terminator.

For construction of gene deletion vectors, upstream (*VdDrs2*-LB, 1, 971 bp) and downstream (*VdDrs2*-RB, 1, 950 bp) fragments of *VdDrs2* gene were amplified using *V. dahliae* genomic DNA as template with paired primers *VdDrs2_LB_-F*/*VdDrs2_LB_-R* and *VdDrs2_RB_*-*F*/*VdDrs2_RB_-R*, respectively. *VdDrs2*_LB_ that contains *Eco*RI sites was cloned into pK2-*HygR* which was digested with *Eco*RI to form pK2-*VdDrs2*_LB_-*HygR* using Exnase MultiS (C112-01, Vazyme, Nanjing, China). Similarly, *VdDrs2*_RB_ PCR product that contains *Xba*I sites was cloned into pK2-*VdDrs2*_LB_-*HygR* which was digested with *Xba*I to generate pK2-*VdDrs2*_LB_*-HygR*-*VdDrs2*_RB_. Similar approach was performed to generate the deletion vectors of *VdNeo1* and *VdP4-4*. The resulting vectors were transformed into *A. tumefaciens* AGL-1 and were subsequently used to transform wild-type *V. dahliae* as described previously (Zhou et al., 2013). Transformants were verified by PCR (primers *VdDrs2-screen-F*/*VdDrs2-screen-R* used for screening Δ*VdDrs2*, primers *VdNeo1-screen-F*/*VdNeo1-screen-R* used for screening Δ*VdNeo1* and primers *VdP4-4-screen-F*/*VdP4-4-R* used for screening Δ*VdP4-4*, Supplementary Table 1).

To construct *VdDnf1* RNAi vector, *VdDnf1*_sense_ (416 bp), and *VdDnf1*_antisense_ (416 bp) strands were generated by PCR with primer pairs *VdDnf1-sense-F*/*VdDnf1-sense-R*, *VdDnf1-antisense-F*/*VdDnf1-antisense-R*. PMBbRNAi was used as an intermediate vector for the construction of transformation vectors (Supplementary Figure 1). The fragment of *VdDnf1*_sense_ that contains *Eco*RI sites was cloned into the modified vector, which was digested with *Eco*RI to generate PMBbRNAi-*VdDnf1*_sense_. Then, the fragment of *VdDnf1*_antisense_ that contains *Bgl*II sites was inserted into PMBbRNAi-*VdDnf1*_sense_ digested with *Bgl*II to generate PMBbRNAi-*VdDnf1*_sense–antisense_, in which the expression of the target fragment was driven by *A. nidulans gpdA* promoter and stopped by *A. nidulans trpC* terminator. Treated the vector with *Xba*I and *Spe*I, and the resultant fragment was inserted into pK2-*HygR*. The resulting vector was transformed into wild-type *V. dahliae* to generate *VdDnf1RNAi* transformant.

To generate complementation vectors, complementary fragments of *VdDrs2* (6,923 bp) containing sequences of promoter (1,670 bp), coding region (4,234 bp), and terminator (1,019 bp) was amplified *via* PCR (primers *VdDrs2-com-F*/*VdDrs2-com-R*, Supplementary Table 1). The resulting PCR products that contains *Hin*dIII site were fused with the upstream sequence of the G418 resistance gene cassette of pK2-*NeoR* which was digested with *Hin*dIII to form pK2-*NeoR*-*VdDrs2* using Exnase MultiS (C113-01, Vazyme, Nanjing, China). The resulting vectors were then transformed into Δ*VdDrs2* and the correct transformants were verified by PCR (primers *VdDrs2-F*/*VdDrs2-R*, Supplementary Table 1). The complemented strain of the *VdDrs2* mutant was abbreviated as Com.

### Pathogenicity assays

Pathogenicity of *V. dahliae* strains was evaluated on Arabidopsis seedlings or cotton seedlings. For Arabidopsis, the assay was performed as described by Wang et al. (2021). Briefly, 3 mL *V. dahliae* spore suspension (1 × 10^7^ spores/ml) was incubated for each pot growing Arabidopsis seedling (4–5 leaves). Arabidopsis seedlings were then grown and maintained in a growth chamber with a 16 h:8 h light-dark cycle at 22°C with 70% relative humidity. The severity of Verticillium wilting symptom was evaluated at the 12 days post-inoculation (dpi). For cotton, seedlings with two true leaves were inoculated by drenching the soil with *V. dahliae* spore suspension (Chen et al., 2021). Each pot was poured with 15 ml *V. dahliae* spore suspension (1 × 10^7^ spores/ml), then transferred into growth chamber at 26°C for 16 h:8 h light-dark cycle. The disease symptom of the plants was evaluated at the 14 dpi. The disease grade was classified as follows: Grade 0 (no symptoms), 1 (0 to 25% wilted leaves), 2 (25 to 50%), 3 (50 to 75%), and 4 (75 to 100%). The disease index was calculated as 100 × [sum (number of plants × disease grade)]/[(total number of plants) × (maximal disease grade)] (Xu et al., 2014). Pathogenicity assays were performed three times with 18 plants for Arabidopsis, and 30 plants for cotton each time.

For pathogenicity detection on cotton leaves, *V. dahliae* spore suspension (1 × 10^7^ spores/ml) was inoculated to cotton leaves by syringe-injection. Each leaf contains four injection sites, and each site was injected with 5 μl spore suspension. Then the cotton leaves were transferred into growth chamber at 26°C for 7 days. The lesion was observed with a stereoscope (V20, ZEISS, Aalen, Germany) and disease damage was evaluated by staining lesions stained with 0.4% trypan blue (Fernández-Bautista et al., 2016). Fungal biomass was estimated by quantitative real-time PCR using *ITS1-F* and *ST-VE1-R* primers as described by Ellendorff et al. (2009). Cotton *GhUBQ7* served as an endogenous plant control. For the relative biomass detection, gDNA was extracted from roots and 3 to 5 cm cotton stems away from the root using plant gDNA kit (Aidlab, Beijing, China), and amplified with primers *ITS1-F/ST-VE1-R* (Supplementary Table 1) for RT-qPCR to detect relative biomass (The reference gene is *GhUBQ7*, GeneBank No. DQ116441).

### Construction of *eGFP*-Vd991 and eGFP-Δ*VdDrs2 V. dahliae* strains, and infection observation

To generate *eGFP* expression cassette, *eGFP* sequence was amplified with paired primers *eGFP-F*(*Not*I)/*eGFP-R*(*Bam*HI) (Supplementary Table 1). The resulting PCR product was inserted into modified PUC-T vector (D2006, Beyotime, Shanghai, China). The gene was placed between the *Beauveria bassiana* constitutive promoter P*gpdB* sequence and *A. nidulans* terminal sequence T*trpC*. Then, the vector was digested with *Xba*I and *Spe*I and inserted into the downstream of the G418 resistance gene cassette of pK2-*NeoR* to form pK2-*NeoR*-P*gpdB*-*eGFP*-T*trpC* using T4 DNA ligase (CV0701, Aidlab, Beijing, China). The resulting vector was transformed into Vd991 or Δ*VdDrs2*, and the correct transformants were selected by fluorescent stereoscope (V20, ZEISS, Aalen, Germany). The fluorescent expression strains were named *eGFP*-Vd991 or eGFP-Δ*VdDrs2*, respectively.

For invasion observation, cotton seeds were soaked in 3‰ of H_2_O_2_ overnight. Then, seeds were covered with one layer of filter paper soaked with water in a 32°C incubator for 2 days. At this time, the radicle undergoes rapid elongation and lateral roots have not emerged. Then the seedlings were inoculated in 50 ml spore suspension (1 × 10^7^ conidia/ml) of *eGFP*-Vd991 or eGFP-Δ*VdDrs2.* At the second day after inoculation, *V. dahliae* hyphae in cotton roots were observed with confocal microscope on the root surface. At the seventh day after inoculation, fragments between 0.5 to 1.5 cm, or 1.5 to 2.5 cm were manually cut out with blade, and then cut the fragments longitudinally. *V. dahliae* hyphae in cotton roots were observed with confocal microscope (Zhao et al., 2014).

### Construction of eGFP::VdDrs2 strains and confocal microscope

The full-length *VdDrs2* sequence was amplified from *V. dahliae* cDNA library with paired primers *VdDrs2-G-F*/*VdDrs2-G-R* (Supplementary Table 1) and *eGFP* was amplified with paired primers *eGFP-F*/*eGFP-R* (Supplementary Table 1). The resulting PCR products were fused by fusion PCR method (Prime STAR, TaKaRa, Kyoto, Japan) with paired primers *eGFP-F*/*VdDrs2-G-R* (Supplementary Table 1). The fused genes were inserted into modified PUC-T vector (D2006, Beyotime) between the *B. bassiana* constitutive promoter P*gpdB* sequence and *A. nidulans* terminal sequence T*trpC* by Exnase MultiS (C112-01, Vazyme, Nanjing, China). Then, the vector was digested with *Xba*I and *Spe*I and inserted into the corresponding sites of pK2-*NeoR* to form pK2-*NeoR*-*eGFP*::*VdDrs2* using T4 DNA ligase (CV0701, Aidlab, Beijing, China). PH^OSBP^ (the pleckstrin homology domain of the human oxysterol binding protein) was widely used as a marker of TGN (Pantazopoulou and Peñalva, 2009). gpdA^mini^-*mRFP*::*PH*^OSBP^ was amplified from plasmid p1793 (Pantazopoulou and Peñalva, 2009) using primers *gpdA^mini^-F/PH-R*. Then the resulting PCR fragment was inserted into pK2-*HygR* to form pK2-*HygR-mRFP*::*PH^OSBP^* by Exnase MultiS (C112-01, Vazyme, Nanjing, China). The resulting vector was transformed into the Vd991 and transformants were selected by fluorescent stereoscope (V20, ZEISS, Aalen, Germany). The vector of pK2-*NeoR*-*eGFP*::*VdDrs2* was transformed into Vd991 expressed TGN marker protein mRFP::PH^OSBP^ or Δ*VdDrs2.* Transformants were selected by fluorescent stereoscope (V20, ZEISS, Aalen, Germany).

Conidial suspensions of resulting strains were inoculated into PDB medium for 3 days. Samples were washed with PBS three times before confocal imaging. For the observation of the fluorescent signal of eGFP, an argon ion laser (Ex = 488 nm, Em = 500 to 530 nm) was used. For the observation of mRFP and FM4-64, the fluorescent signal was acquired using a He-Ne laser (Ex = 552 nm, Em = 570 to 670 nm). Finally, confocal images were captured with confocal microscope (Leica SP8, Wetzlar, Germany).

### Phenotypic assays

*V. dahliae* growth and nutrition (carbon/nitrogen) assays were referred to Li et al. (2020). Briefly, conidia were collected from 10 days old PDA cultures and the spores was cultured on CZA plates (3 μl 1 × 10^7^ conidia/ml for each plate) or on the modified CZA in which 0.3% sucrose was replace with glucose, dextrose, maltose, fructose, galactose, trehalose, mannitol, xylitol, sorbitol, or starch; or 0.3% NaNO_3_ was replaced by NaNO_2_, peptone, (NH_4_)_2_SO_4_, and Co(NH_2_)_2_, respectively. Plates were incubated at 26*^o^*C for 7 days. All the experiments were performed in triplicate and repeated thrice with different batches of conidia. Colony growth was evaluated *via* measuring colony diameter.

### Biomass assay and conidial yield determination of *V. dahliae*

For biomasses assay, conidial suspension of the strains was inoculated into PDB medium. The final concentration of conidia in the PDB medium was 1 × 10^4^ conidia/ml, and then the conidia suspension was inoculated at 26°C on a rotary shaker (200 rpm). The biomasses were measured at the 4 dpi. Mycelia were harvested and dried by Vacuum freeze dryer (Martin Christ, Osterode am Harz, Germany) and weighted by Mettler Toledo XS105 (Switzerland). Biomass relative to Vd991 = Biomass (Vd991, Δ*VdDrs2*, or Com)/Biomass of Vd991. For conidia yield assay, fungal spores were added into PDA (temperature bellow 60°C) to the final concentration of 5 × 10^4^ conidia/ml. Plates were placed at 26^°^C for 10 days. Conidia were collected with a puncher and suspended in water with 0.05% Tween 80. Conidial concentration was determined by hemocytometer. All experiments were performed in triplicates with three independent repeats.

### Conidial germination assay of *V. dahliae*

Conidial suspension (100 μl, 1 × 10^7^ conidia/ml) was inoculated on PDA plates (90 mm) and cultured at 26°C for 8 h. The conidial germination was microscopically observed every hour until all the conidia have geminated.

### Scanning electron microscopy

To observe the microsclerotia formation, Vd991, Δ*VdDrs2*, and Com were cultured for 2 weeks on the CZA plates as described previously (Xiong et al., 2019). The colony samples were directly imaged with a scanning electron microscope S-3400N (Hitachi, Tokyo, Japan).

### High-performance liquid chromatography analysis of metabolites

High-performance liquid chromatography (HPLC) analysis of metabolites was performed as described by Spadaro et al. (2020) with minor modifications. Strains (Vd991 or Δ*VdDrs2*) were cultured on PDA plates for 7 days. The conidia were collected and inoculated into CZB medium at a final concentration of 1 × 10^4^ conidia/ml. The culture was then maintained at 200 rpm for 14 days. A total of three liters of culture were prepared for each strain. The cells were collected by centrifugation, and dried in a vacuum freeze dryer (Martin Christ, Osterode am Harz, Germany). The dry weight of Vd991 and Δ*VdDrs2* was then determined with a Mettler Toledo XS105 (Switzerland). In the meantime, the supernatants were concentrated in proportion to the dry weight of each strain using N-1100 rotary evaporator (EYELA, Tokyo, Japan). HPLC was performed using a ZORBAX SB-C18 (4.6 × 250 mm, 5 μm, Agilent, Santa Clara, CA, United States) under a flow of 1 ml/min with PDA detector SPD-M20A (Shimadzu, Kyoto, Japan). Solvent A was H_2_O acidified with 0.01% formic acid, while solvent B was methanol. Elution gradient started with 10% of solvent B and maintained for 10 min, increased to 100% methanol in 50 min, then reduced to 10% methanol in 20 min. The sample volume was 10 μl. For the detection of known toxins, sulfacetamide (SFA) (Sigma-Aldrich, Saint Louis, MO, United States) and fumonisin B1 (FB1) (Sigma-Aldrich, Saint Louis, MO, United States) were used as authentic standards.

### Pathogenicity detection of crude toxins

The crude toxin preparation is made by condensing the fermentation broth through Concentrator (N-1100 rotary evaporator, EYELA, Tokyo, Japan). Three liters fermentation broth was condensed into 100 ml that was used as crude toxins. 5 μl crude toxin preparation was injected on a cotton leaf (four inoculation sites per leaf). Then, the leaves transferred into growth chamber at 26°C for 3 days. The disease damage was evaluated by staining lesions with 0.4% trypan blue.

### Data analysis

Statistical analyses of the data were performed using GraphPad prism. Standard errors were calculated using SPSS Statistics software (SPSS Inc., Chicago, IL, United States). Multiple groups of data were compared using ANOVA with a LSD multiple comparisons test in SPSS. Two groups of data were compared using *t* tests in SPSS.

## Results

### Disruption of VdDrs2 leads to a significant decrease in *V. dahliae* virulence

**FIGURE 1:**
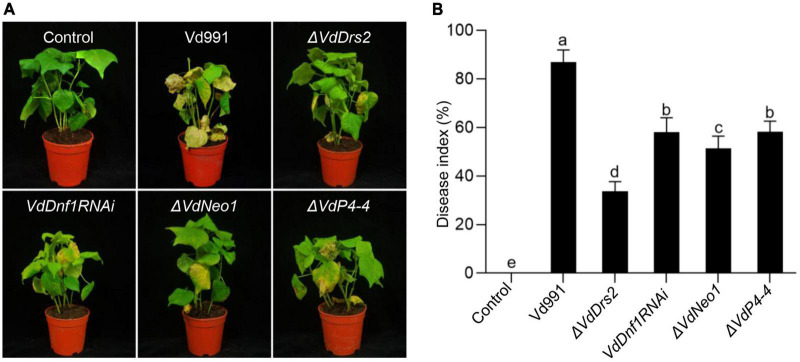
Knock out of *VdDrs2*, *VdNeo1*, and *VdP4-4*, or knock down of *VdDnf1* in *V. dahliae* reduces the pathogenicity to cotton. **(A)** Wilt symptom of cotton plants inoculated with spores of knock-out stains (Δ*VdDrs2*, Δ*VdNeo1*, and Δ*VdP4-4*), knock-down strain (*VdDnf1RNAi*), and wild type strain (Vd991) by root-dipping method. Control, cotton seedlings were inoculated with sterile water. **(B)** Disease index of cotton plants inoculated with *V. dahliae* spores of mutant strains and wild type strain. The grades of disease symptoms are described in the methods. 30 cotton plants were used per treatment. Different letters (a, b, c, d, and e) indicate a significant difference (*P* < 0.05) based on one-way ANOVA with Tukey multiple comparisons test.

Four candidate genes (VDAG_06574, VDAG_06743, VDAG_09020, and VDAG_00595) were obtained by BLAST search of the *V. dahliae* genome using the amino acid sequences of the *Saccharomyces cerevisiae* flippase proteins Dnf1, Dnf2, Drs2, Dnf3, and Neo1.^1^ We renamed the genes as VdDrs2, VdDnf1, VdNeo1, and VdP4-4, respectively, with reference to phylogenetic analysis and nomenclature in yeast. Phylogenetic analysis suggests that these putative flippases from *V. dahliae* could be classified into four subgroups with other fungi, including *B. bassiana, S. cerevisiae*, *Fusarium graminearum*, *M. oryzae*, and *Neurospora crassa* (Supplementary Figure 2). To investigate the role of the P4-ATPases in *V. dahliae* pathogenicity, *VdDrs2*, *VdNeo1*, and *VdP4-4* were knocked out in *V. dahliae* strain 991 by homologous recombination (Supplementary Figures 3A–C). Due to the unavailability of *VdDnf1* knock-out mutant, we alternatively knocked down the gene by RNAi (Supplementary Figures 3D,E). We then challenged cotton plants with the resulting strains. Loss of function of *VdDrs2*, *VdNeo1*, or *VdP4-4*, and *VdDnf1* RNAi significantly decreased the pathogenicity of the pathogen in cotton. Among *VdP4-ATPase* mutants, the greatest decrease in pathogenicity was observed in Δ*VdDrs2* (Figures 1A,B).

*VdDrs2* (annotated as VDAG_06574 in the genome of VdLs.17) contains a 4,128 bp cDNA sequence. The protein sequence homology between VdDrs2 and ScDrs2 reaches 59.74%. The *VdDrs2* gene encodes a putative protein of 1,375 amino acids with ten transmembrane regions, N-terminal regions (1–306) and C-terminal regions (1286–1376) (Supplementary Figures 4A,B).

**FIGURE 2:**
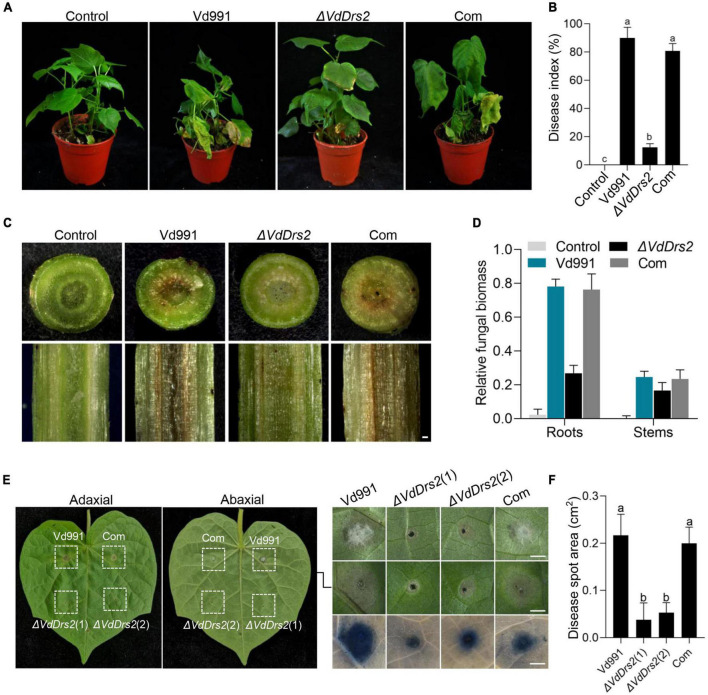
Knockout of *VdDrs2* reduces the pathogenicity of *V. dahliae* to cotton. **(A)** Disease symptoms of cotton plants inoculated with spores of Vd991, Δ*VdDrs2*, and complemented strains (Com) by root-dipping inoculation. Control, cotton seedlings were inoculated with sterile water; Vd991, Δ*VdDrs2*, and Com, seedlings were inoculated with spore suspensions (5 ml, 1 × 10^7^ spores/ml) of wild type strain Vd991, Δ*VdDrs2*, and Com, respectively. **(B)** Disease index of infected cotton plants. The grades of disease symptoms are described in the methods. 30 cotton plants were used per treatment. Different letters (a, b, and c) indicate a significant difference (*P* < 0.05) based on one-way ANOVA with Tukey multiple comparisons test. **(C)** Comparison of vascular discoloration associated with VW in cotton plants infected with Vd991, Δ*VdDrs2*, and Com strains by root-dipping inoculation. Top, transverse sections of the infected cotton stems; bottom, longitudinal sections of the infected cotton stems. Scale bar represents 0.2 mm. **(D)** Fungal biomass in cotton plants 14 days post root-dipping inoculation. Fungal biomass was determined by quantitative real-time PCR using *ITS1-F* and *ST-VE1-R* primers as described by Ellendorff et al. (2009). Cotton *GhUBQ7* served as an endogenous plant control. **(E)** Disease symptoms of cotton leaves after inoculation with Vd991, Δ*VdDrs2*, and Com spores. Each leaf had four injection spots. 5 μl conidia solution (1 × 10^7^ spores/ml) was used for each injection. Left column, adaxial leaves; middle column, abaxial leaves; right column, enlargement, and trypan blue staining of lesion regions in the middle column. Trypan blue staining was used to detect the cytolytic area of the lesion regions (bottom). Δ*VdDrs2*(1) and Δ*VdDrs2*(2) are technical replicates. Scale bars represent 0.2 cm. **(F)** Statistics of lesion area on the leaves inoculated with Vd991, Δ*VdDrs2*, and Com.

To confirm the role of *VdDrs2* in pathogenicity, a complemented strain was generated. Root dip infection assay showed that VW symptom caused by Δ*VdDrs2* was significantly less severe than that by the wild type and complemented strain (Figures 2A,B). Browning of the xylem is a typical VW symptom in cotton. The browning degree in the stems of the cotton infected by the disrupted strain was much less than that of the wild type and complemented strain (Figure 2C). The fungal biomass in the infected cotton plants was estimated by quantitative real-time PCR (RT-qPCR). The amount of *V. dahliae* DNA was significantly lower in the Δ*VdDrs2*-infected plants than that in wild type-infected plants and complemented strain-infected plants (Figure 2D). The disease index of the cotton infected with Δ*VdDrs2* was reduced by 53.17% compared with the wild type (Figure 2B). Injecting the pathogen spores in cotton leaves, the infected area produced by Δ*VdDrs2* strain was significantly smaller than that by the wild type and complemented strain (Figures 2E,F). Same decreased severity was observed in Arabidopsis infected with Δ*VdDrs2* (Supplementary Figures 5A,B).

**FIGURE 3:**
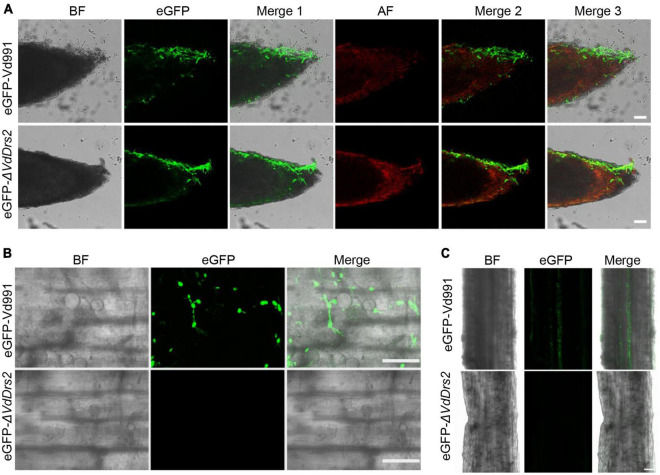
Knockout of *VdDrs2* delays the infection of *V. dahliae* to cotton. **(A)** Confocal microscopy images of eGFP-labeled hyphae on the root surface. Cotton roots were infected with *eGFP*-Vd991 and eGFP-Δ*VdDrs2* by pouring 50 mL spore suspension (1 × 10^7^ spores/ml) to each growth pot. Root tips were observed with confocal microscope 2 days after inoculation. Scale bars represent 50 μm. BF, bright field; AF, autofluorescence; Merge 1, the green fluorescence image is merged with bright field image; Merge 2, the green fluorescence image is merged with red fluorescence image; Merge 3, the green fluorescence image is merged with bright field image and red fluorescence image. **(B)** Confocal microscopy images of eGFP-labeled hyphae in the roots. Cotton roots were infected with *eGFP*-Vd991 and eGFP-Δ*VdDrs2* for 7 days, respectively. Fragments between 0.5 to 1.5 cm away from the root tip were cut out for section and were observed with confocal microscope. Scale bars represent 25 μm. **(C)** The penetrating hyphae of *eGFP*-Vd991 or eGFP-Δ*VdDrs2* strains were observed by confocal microscope. Cotton roots were infected with *eGFP*-Vd991 and eGFP-Δ*VdDrs2* for 7 days. Fragments 1.5–2.5 cm away from the root tip was cut into thin slices and were observed with confocal microscope. Scale bar represents 50 μm.

To observe the invading hyphae of *V. dahliae* in cotton tissues, we infected the hydroponic seedlings of cotton with *eGFP*-Vd991 and eGFP-Δ*VdDrs2*, respectively. At the second day after inoculation, the fluorescence-labeled hyphae of both *eGFP*-Vd991 and eGFP-Δ*VdDrs2* were observable on the root surface (Figure 3A). At the seventh day after inoculation, eGFP-labeled hyphae were observed in the root infected by *eGFP*-Vd991 strain, while no significant eGFP signal was detected in the root infected by the eGFP-Δ*VdDrs2* strain (Figures 3B,C), indicating that the disruption of *VdDrs2* significantly retards the invasion of plant cells by the fungus.

### VdDrs2 is localized to plasma membrane, vacuoles, and *trans*-Golgi network

**FIGURE 4:**
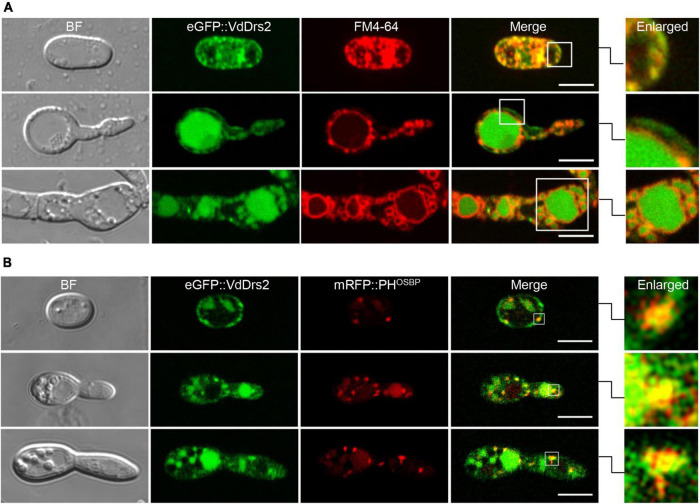
VdDrs2 is localized to plasma membrane, vacuoles and *trans*-Golgi network (TGN). **(A)** Confocal imaging of eGFP::VdDrs2 (green, Ex = 488 nm, Em = 500–530 nm) in different growth stages of *V. dahliae*. The plasma membrane and vacuoles were stained by FM4-64. BF, bright field; Merge, the green fluorescence image is merged with red fluorescence image; Enlarged, each boxed area on the right is enlarged from the white box in merge. Scale bars represent 5 μm. The images were captured with confocal microscope (Leica SP8, Wetzlar, Germany). **(B)** VdDrs2 and PH^OSBP^ were colocalized in cells at different growth stages of *V. dahliae*. PH^OSBP^, the pleckstrin homology domain of the human oxysterol binding protein, was used to indicate the location of TGN. BF, bright field; Merge, the green fluorescence image is merged with red fluorescence image; Enlarged, each boxed area on the right is enlarged from the white box in merge. Scale bars represent 5 μm.

To observe the subcellular localization of VdDrs2, the gene was fused with *eGFP* gene. The eGFP::VdDrs2 fusion protein was expressed in the Δ*VdDrs2* mutant. The expression of eGFP::VdDrs2 could recover *V. dahliae* pathogenicity to cotton (Supplementary Figures 6A–D), indicating that the fusion did not impair the function of VdDrs2. Confocal observation showed that VdDrs2 was localized to plasma membrane and vacuoles (Figure 4A). PH^OSBP^ was widely used as a marker of TGN (Pantazopoulou and Peñalva, 2009). In the strain expressing eGFP::VdDrs2 fusion protein and PH^OSBP^::mRFP fusion protein, the eGFP and mRFP signals were co-localized in the puncta that scattered in the cell (Figure 4B).

### Disruption of VdDrs2 decreases the biomass and sporulation, and impairs the microsclerotia formation of *V. dahliae*

**FIGURE 5:**
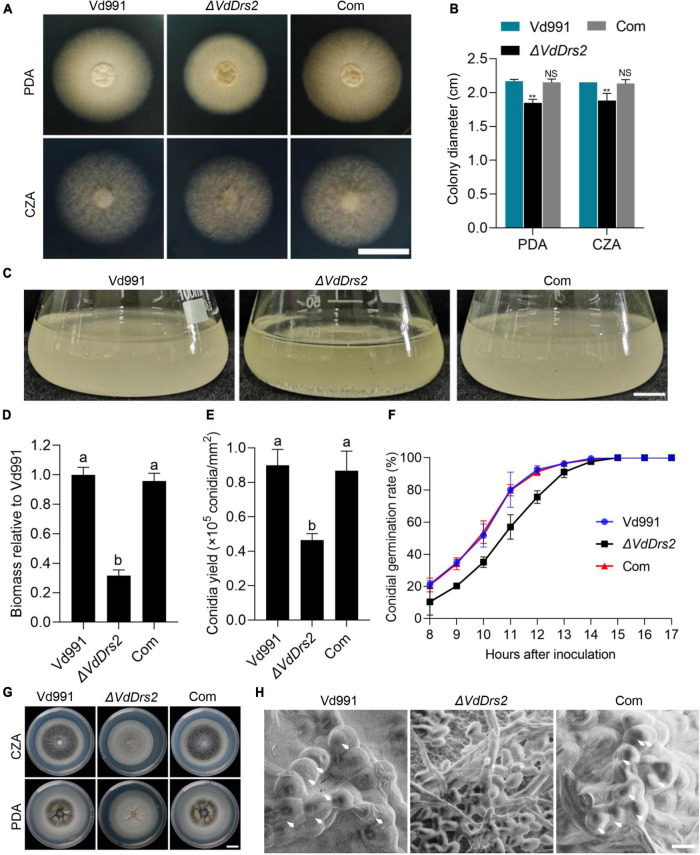
Disruption of VdDrs2 decreases the colony growth, biomasses, sporulation, and microsclerotia formation and delays conidia germination. **(A,B)** Disruption of *VdDrs2* decreased the growth of *V. dahliae*. Vd991, wild type strain; Δ*VdDrs2*, *VdDrs2* knocked-out strain; Com, *VdDrs2*-complemented strain. The strains were cultivated on PDA and CZA plates at 26°C for 7 days. Scale bar represents 1 cm. Two groups of data were compared using *t* tests in SPSS. **P < 0.01, NS: not significant. **(C,D)** Disruption of *VdDrs2* reduced biomass of *V. dahliae*. Scale bar represents 1 cm. Different letters (a, b) indicate a significant difference (*P* < 0.05) based on one-way ANOVA with Tukey multiple comparisons test. **(E)** Knockout of *VdDrs2* reduces the conidial yields of *V. dahliae*. Different letters (a, b) indicate a significant difference (*P* < 0.05) based on one-way ANOVA with Tukey multiple comparisons test. **(F)** Knockout of *VdDrs2* delayed conidial germination of *V. dahliae*. Conidia were cultivated onto PDA plates, and the germination rate was counted hourly from 8th hours until all the conidia have germinated. Each strain assay was performed thrice. The error bars indicate standard deviations from three repeats of the experiment. **(G)** Knockout of *VdDrs2* impaired melanin production. Vd991, Δ*VdDrs2*, and Com strains were cultivated on PDA and CZA plates at 26°C for 14 days. Scale bar represents 1 cm. **(H)** Scanning electron microscope of microsclerotia formation. White arrows indicate microsclerotia. Scale bar represents 5 μm.

With various carbohydrate and nitrogen sources, the Δ*VdDrs2* strain exhibited decreased colony growth and biomass, compared with the wild type (Figures 5A–D and Supplementary Figures 7A–D). The conidia yield of Δ*VdDrs2* strain was significantly reduced (Figure 5E), and the conidial germination rate of the VdDrs2-disrupted strain was lower than that of the wild type control and the complemented strain within 14 h after incubation (Figure 5F). Noticeably, melanin synthesis was severely disrupted in Δ*VdDrs2* strain (Figure 5G). No microsclerotia was found in Δ*VdDrs2* strain (Figure 5H).

### Disruption of VdDrs2 decreases the secretion of *V. dahliae* toxins

**FIGURE 6:**
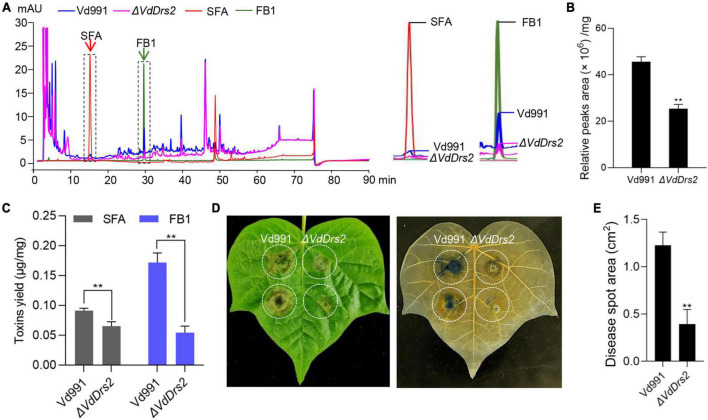
Disruption of VdDrs2 decreases the amount of secondary metabolites in fermentation broth of *V. dahliae* and reduced the toxicity of the crude extraction to cotton leaves. **(A)** High-performance liquid chromatography (HPLC) profile of metabolites from Vd991 and Δ*VdDrs2*. Authentic sulfacetamide (SFA) and fumonisin B1 (FB) were included to show the presence of these compounds in the fermentation broths. SFA, sulfacetamide; FB1, fumonisin B1. **(B)** Total peaks area of metabolites produced per mg fungi to show Δ*VdDrs2* fermentation were lower than those of Vd991. **(C)** Production of SFA and FB1 was decreased in Δ*VdDrs2* compared to Vd991. The amount of the each toxin produced by one mg fungi was determined with standard curve of SFA and FB1 (μg toxin/mg fungi). **P < 0.01. The value is the mean of three separate determinations. **(D)** Disease symptoms of cotton leaves after inoculation with crude toxins produced by Vd991 and Δ*VdDrs2*. Trypan blue staining was used to detect area of cytolysis (right). **(E)** Statistical analysis of the area of toxin-poisoned tissue per square centimeter. cm^2^: per square centimeter. Disease spot area was measured using ImageJ software. Two groups of data were compared using *t* tests in SPSS. **P < 0.01. All experiments were performed in triplicate.

Toxins are regarded as important virulence factors that contribute to VW disease (Klosterman et al., 2011; Lo Presti et al., 2015; Ma et al., 2021), and P4-ATPases have been reported involved in vesicle-mediated secretory and endocytic pathways (Walter et al., 2002; Gilbert et al., 2006; Yun et al., 2020). HPLC assay showed that the peak profile of the Δ*VdDrs2* fermentation broth was different with the wild type: both the peaks height and peaks area of the Δ*VdDrs2* fermentation broth were lower than those of the wild type, showing that a decrease in metabolites secreted by knockout strain (Figures 6A,B). As there are two toxins, SFA and FB1, identified in *V. dahliae* (Zhang et al., 2016; Xu et al., 2022), we quantified the amount of these two toxins, in the fermentation broths of Vd991 and Δ*VdDrs2*. The amounts of these two VW toxins produced by one mg of Δ*VdDrs2* mycelia were significantly lower than that by wild type (Figure 6C). Injecting cotton leaves with crude preparation containing VW toxins, the size of necrosis area caused by the crude preparation from Δ*VdDrs2* culture solution was much smaller than that from the wild type control (Figures 6D,E), indicating the decreased toxicity of the Δ*VdDrs2* fermentation broth to the cells.

## Discussion

Microsclerotia are the resting propagule of *V. dahliae*. Microsclerotia can survive in soil for many years (Pegg and Brady, 2002). This long-term survival in nature makes the soil born *V. dahliae* difficult to control. It has been known that melanin is indispensible for microsclerotia survival and contributes to the resistance of microsclerotia to environmental stresses (Jacobson, 2000; Shang et al., 2013; Li J. J. et al., 2019; Fan et al., 2020; Harting et al., 2020). Emerging evidences suggest the correlation of melanin biosynthesis and microsclerotia formation with virulence of *V. dahliae* (Xiong et al., 2015; Tian et al., 2016; Zhang et al., 2017; Wang et al., 2018; Yu et al., 2019; Zheng et al., 2019). Our results reveal a key role of VdDrs2 in microsclerotia formation and melanin production, which contributes to the pathogenicity of *V. dahliae*. However, how the P4-ATPases and P4 ATPases-associated vesicle transport participate in microsclerotia formation and melanin production requires further study.

*V. dahliae* is considered as a hemibiotrophic pathogen (Kemen and Jones, 2012; Buhtz et al., 2015; Narváez et al., 2020). At the biotrophic phase, the pathogen generates low level of toxins to suppress PCD (Programmed cell death) to facilitate its colonization and infection. During necrotrophic phase, it produces high level of toxins to stimulate PCD to facilitate the necrotrophic fungal growth (Tian et al., 2010). Thus, mycotoxins are regarded as the key virulence factors of VW diseases (Gunupuru et al., 2017). In this study, we show that VdDrs2 is involved in the toxin secretion of *V. dahliae* and the disruption of *VdDrs2* gene significantly reduces the secretion of *V. dahliae* toxins which is able to cause Verticillium wilting symptom (Figures 6A–E); the loss-of-VdDrs2 function mutation significantly impairs the virulence of *V. dahliae* (Figure 2). Therefore, our data provide an evidence for the “toxin theory”.

P4-ATPases have been reported to be associated with the virulence of phytopathogenic fungi (Balhadère and Talbot, 2001; Gilbert et al., 2006; Li B. et al., 2019; Yun et al., 2020). Loss-of-function of MgATP2, a *M. oryzae* P4-ATPase, disrupted the secretion of extracellular enzymes and thus decreased its pathogenicity to rice (Gilbert et al., 2006). Yun et al. (2020) identified five P4-ATPases (FgDnfA, FgDnfB, FgDnfC1, FgDnfC2, and FgDnfD) in *F. graminearum*, and showed that the disruption of FgDnfA and FgDnfD remarkably decreased the secretion of mycotoxin deoxynivalenol (DON) and significantly impaired the pathogenicity of the fungus to wheat. In this study, we identified four genes encoding P4-ATPases in *V. dahliae* genome could be classified into four subgroups (Supplementary Figure 2). We show that knock-out of *VdDrs2*, *VdNeo1*, and *VdP4-4*, or knock down of *VdDnf1* can decrease the virulence of *V. dahliae* in various degrees (Figure 1). Of them, the deletion of VdDrs2 leads to the largest reduction in virulence. These data suggest an important role of VdDrs2 in the virulence of the pathogen. Our HPLC results further shows that the production capacity of the loss of function of VdDrs2 hyphae for the generation of two known VW toxins, SFA, and FB1, are significant decreased (Figure 6C), suggesting that VdDrs2 is implicated in the secretion of VW toxins. Whether the three other P4-ATPases, i.e., VdNeo1 VdP4-4, and VdDnf1, are participated in the toxin secretion needs to be investigated.

VdDrs2 shows 59.74% homology to yeast ScDrs2. Disruption of ScDrs2 in yeast lead to retention of secretory vesicles within cells, indicating a role of the protein in clathrin-mediated exocytosis (Walter et al., 2002). The deletion of *VdDrs2* significantly decreased the amount of secondary metabolites and specific VW toxins, SFA, and FB1, in fermentation solution and reduced the toxicity of the liquor to cotton leaves (Figures 6A–E), suggesting an important role of VdDrs2 in mycotoxin secretion. ScDrs2 is reported to be localized to plasma membrane, EE (early endosome), and TGN, and involved in the transport between plasma membrane to TGN, or TGN to EE (Chen et al., 1999; Gall et al., 2002; Hua et al., 2002; Alder-Baerens et al., 2006; Liu et al., 2007). However, the journey of ScDrs2-mediated transport does not include EE to vacuoles (Graham, 2004). In this study, we observed a significant signal of eGFP:VdDrs2 in plasma membrane and TGN, as well as vacuoles (Figures 4A,B). Vacuole is a place where many secondary metabolites are synthesized or stored (Keller, 2015). The vacuole location of VdDrs2 suggests that the VdDrs2-associated toxin secretion pathway includes the journey from vacuoles to plasma membrane. However, details of the P4 ATPases-associated exocytosis of mycotoxins await further investigations.

Our recent study demonstrated that overexpression of Arabidopsis P4-ATPase *AtALA7* gene could promote the transport of a VW toxin from plasma membrane to vacuoles for detoxification by compartmentation, thus significantly increasing the resistance of transgenic plants against VW (Wang et al., 2021). In the present study, we further indicate a key role of *V. dahliae* P4-ATPases in the pathogenicity of VW disease. Therefore, A dual strategy: promoting the detoxification of host plants while impairing the secretion of mycotoxins produced by the pathogen, can be designed for the successful control of this recalcitrant pathogen.

## Data availability statement

The original contributions presented in this study are included in the article/Supplementary material, the VdDrs2 sequence data presented in the study are deposited in the Genbank repository, accession number ON661561, further inquiries can be directed to the corresponding author.

## Author contributions

YP conceived, supervised the project, and wrote the manuscript. HR performed gene knock out in *V. dahliae*; phenotypic observation, toxicity and pathogenicity assays, data analysis, and wrote the manuscript. XL, LW, and YC performed pathogenicity assays on cotton. YL participated in the construction of gene knockout vectors and mutant screening. ML and JS participated in phenotypic assays. FW participated in pathogenicity assays on Arabidopsis. JZ participated in HPLC experiments. XY performed scanning electron microscope. YF and DJ participated in data processing. All authors contributed to the article and approved the submitted version.

## Footnotes

1
https://blast.ncbi.nlm.nih.gov/Blast.cgi
