# Supplementary material for: Loss of function of VdDrs2, a P4-ATPase, impairs the toxin secretion and microsclerotia formation, and decreases the pathogenicity of Verticillium dahliae
Source: Front Plant Sci. 2022 Aug 22;13:944364. doi: 10.3389/fpls.2022.944364 (PMC9443849; doi:10.3389/fpls.2022.944364)
Supplement: Supplementary file 1 [file Data_Sheet_1.docx]

Supplementary Material

# Supplementary Figures and Tables

Supplementary Figure 1. Vector infographic of pK2-*HygR*, pK2-*NeoR* and pMBbRNAi.

Supplementary Figure 2. Neighbor-joining phylogenetic tree of P4 ATPases.

Supplementary Figure 3. Construction strategy and identification of knockout or downregulation of *P4 ATPases* in *V. dahliae*.

Supplementary Figure 4. Gene and structure of VdDrs2.

Supplementary Figure 5. Knockout of *VdDrs2* reduces the pathogenicity of *V. dahliae* to Arabidopsis.

Supplementary Figure 6. Fusion of eGFP does not impair the function of VdDrs2 in pathogenicity of *V. dahliae*.

Supplementary Figure 7. Colony morphology of the different strains on the media with various carbon and nitrogen source.

Supplementary Table 1. Primers used in this study.


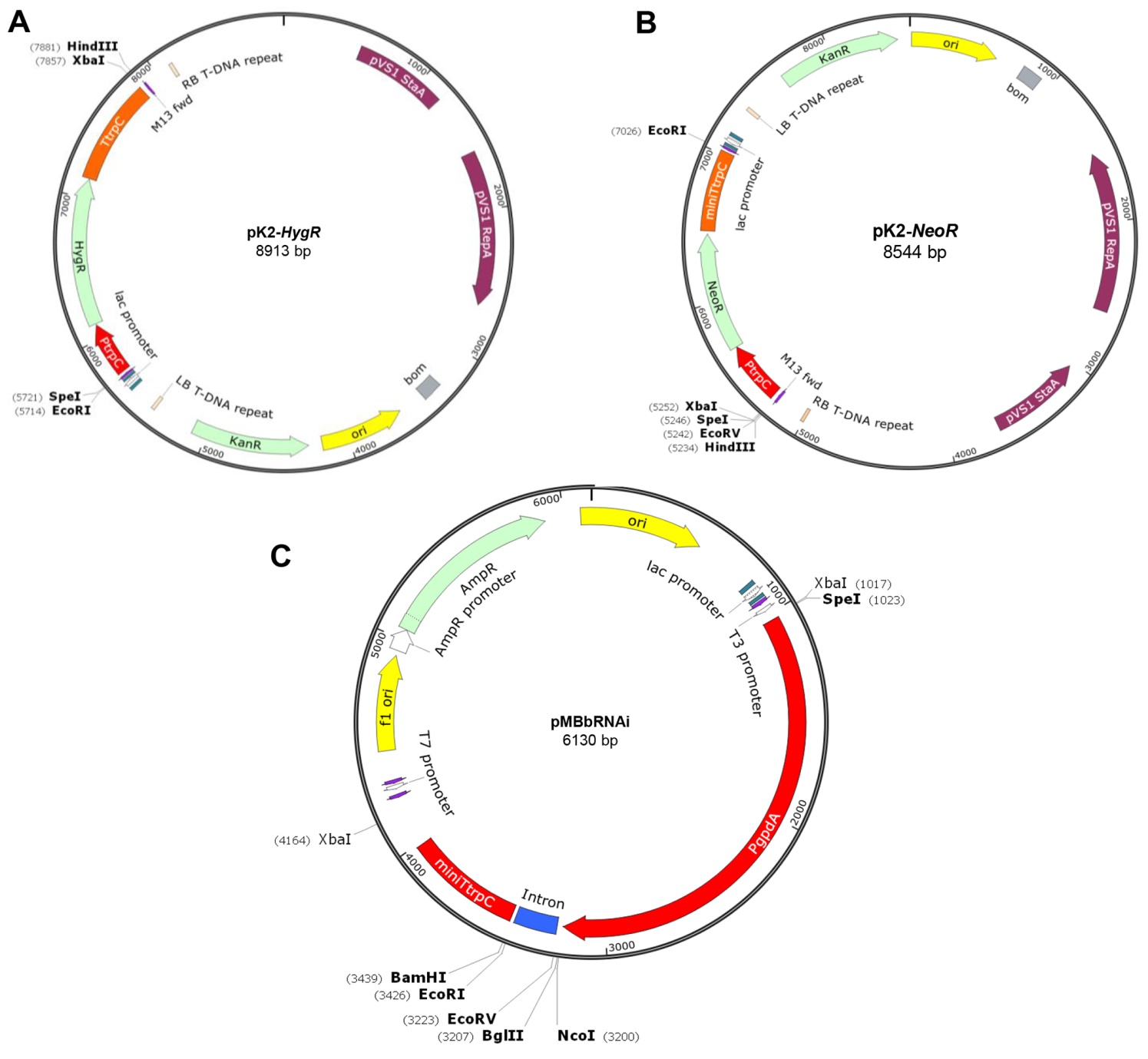
**Supplementary Figure 1.** Vector infographic of pK2-*HygR*, pK2-*NeoR* and pMBbRNAi. (**A**) pK2-*HygR*. (**B**) pK2-*NeoR*. (**C**) pMBbRNAi.


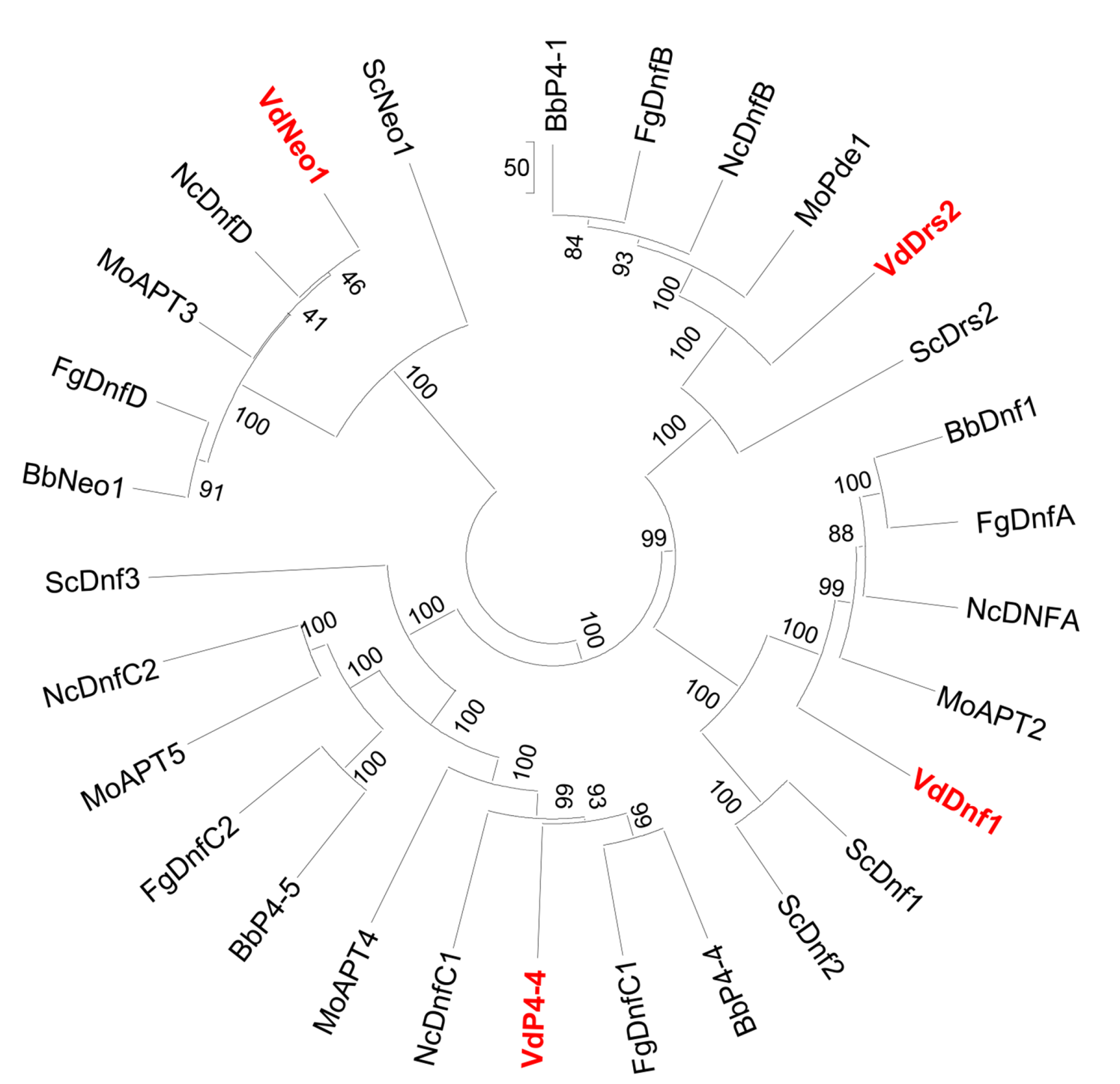
**Supplementary Figure 2.** Neighbor-joining phylogenetic tree of P4 ATPases. Vd*, Verticillium dahliae*; Bb, *Beauveria bassiana*; Sc*, Saccharomyces cerevisiae*; Fg, *Fusarium graminearum*; Mg, *Magnaporthe oryzae*; Nc, *Neurospora crassa*. Bars (= 50) represent sequence divergence. Cluster analysis was conducted using MEGA version 5.05. Branch lengths are indicated in a circletree.

**Supplementary Figure 3.**
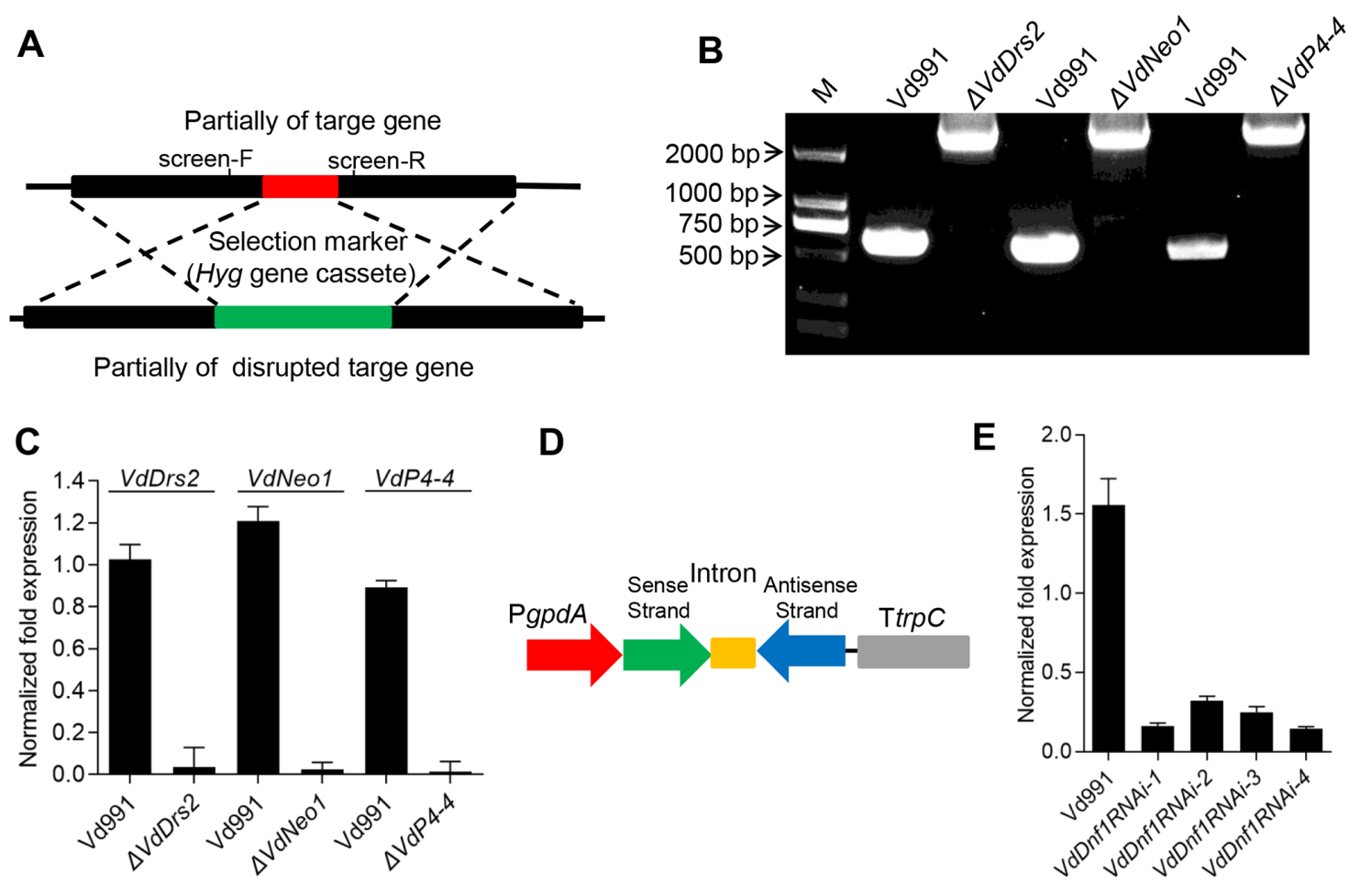
 Construction strategy and identification of knockout or downregulation of *P4 ATPases* in *V. dahliae*. (**A**) Schematic model of construction for *P4 ATPases* replacement vectors. (**B**) PCR identification analysis of Δ*VdDrs2*, Δ*VdNeo1* and Δ*VdP4-4* strains. M: 2000 DNA ladder (TIANGEN, Beijing, China). (**C**) Expression levels of P4 ATPases genes in wild-type (Vd991), *P4 ATPases* knock-out mutants (Δ*VdDrs2*, Δ*VdNeo1* and Δ*VdP4-4*). (**D**) Schematic model of construction for *VdDnf1* RNA interference vector. (**E**) Expression levels of *VdDnf1* gene in wild-type (Vd991), *VdDnf1* downregulation mutants (*VdDnf1RNAi-1*, *VdDnf1RNAi-2, VdDnf1RNAi-3* and *VdDnf1RNAi-4*). RNA extraction and gene expression For gene transcription assays, *V. dahliae* strains were cultured in PDB (broth) medium for 72 h (200 rpm, 26℃) and hyphae were harvested for RNA extraction. RNA concentration was measured by NanoPhotometer (N50 Touch, Implen), and RNA was reverse-transcribed using PrimeScript RT reagent Kit (RR037A, TaKaRa, Kyoto, Japan). RT-qPCR was performed in CFX96 Real-Time System (Bio-Rad). PCR reaction mixtures (20 μl final volume) were as follows: 10 μl iQSYBR Green Supermix (1708882, Bio-Rad), 0.5 μM forward and reverse primers, 100 ng cDNA template. *VdGAPDH* (GenBank no. XM_009656719) and *β-tubulin* (GenBank no. XM_009651338) were used as the internal reference. The regular PCR cycling conditions were as follows: 95℃, 3 min; followed by 40 cycles of 95℃, 10 s; 55℃, 30 s and 72℃, 30 s. Relative expression of the target genes was normalized to the quantity of the reference gene (normalized fold expression) and processed in CFX Manager 3.1 software (BioRad). The assays were performed in triplicate. Error bars indicate one standard deviation. Primers used for RT-qPCR analysis are listed in Supplementary Table 1.

**
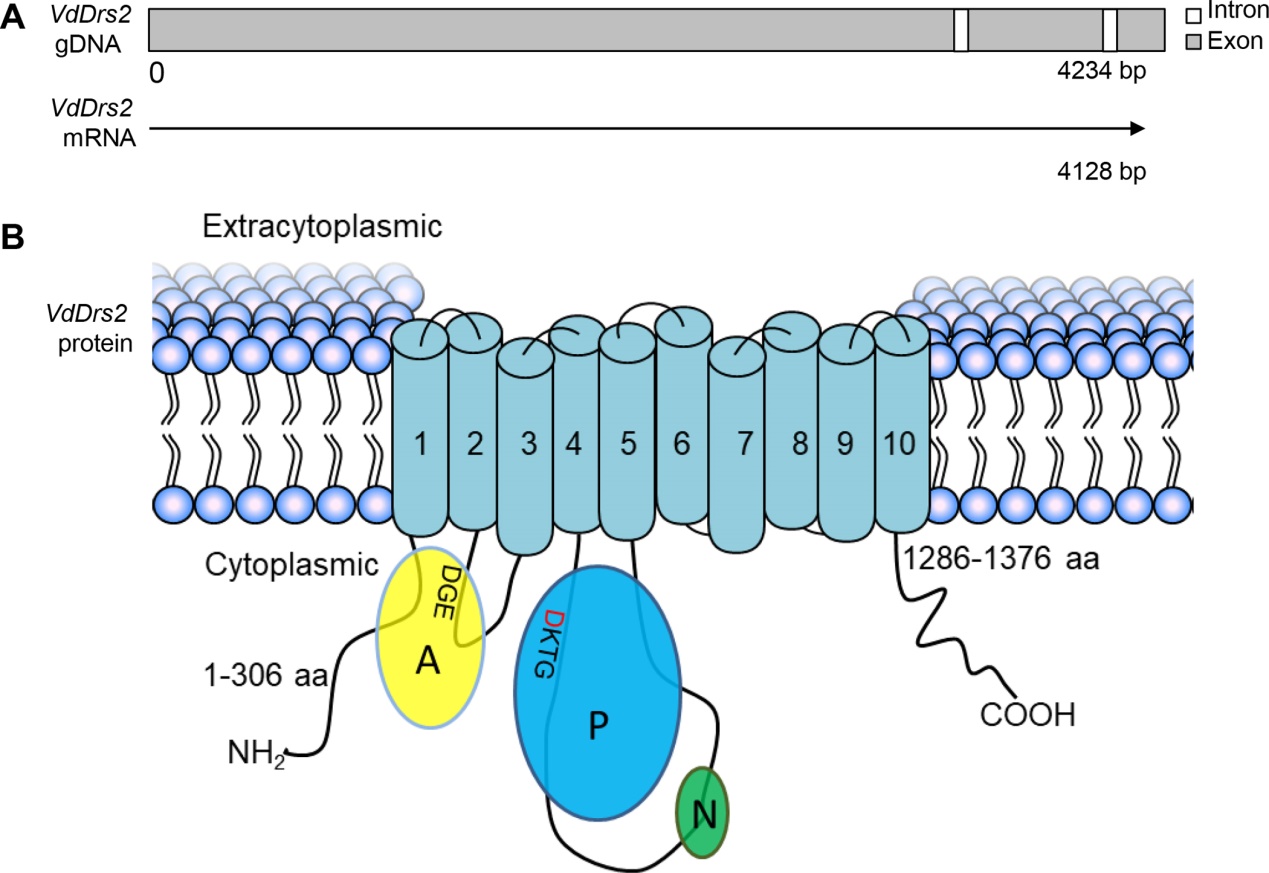
Supplementary Figure 4.** Gene and structure of VdDrs2. **(A)** Intron and exon of *VdDrs2* gene. Genomic DNA and cDNAs were compared using MegAlign. Introns and exons are marked in white and grey, respectively. **(B)** Schematic overview of the structural organization of VdDrs2 protein. A, actuator domain; DGE, Asp-Gly-Glu; N, nucleotide binding domain; P, phosphorylation domain; DKTG, Asp-Lys-Thr-Gly; 1-10: transmembrane-spanning segments. Predicted topological model of VdDrs2. The transmembrane regions were predicted by using the TM predict program, https://services.healthtech.dtu.dk/service.php?TMHMM-2.0 (2021).

**
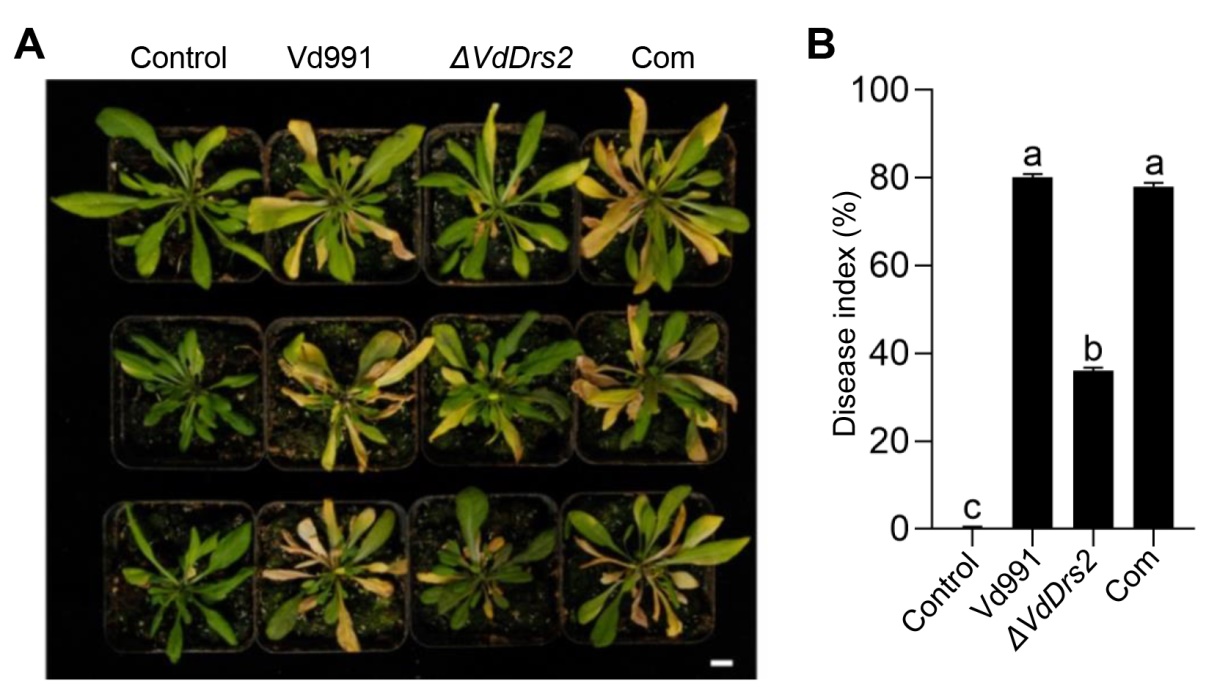
Supplementary Figure 5.** Knockout of *VdDrs2* reduces the pathogenicity of *V. dahliae* to Arabidopsis. (**A**) Disease symptoms of Arabidopsis plants inoculated with water and spores of Vd991, Δ*VdDrs2* and complemented strain (Com). Scale bar represents 1 cm. (**B**) Disease index of Arabidopsis plants infected by water, Vd991, Δ*VdDrs2* and Com. Eighteen Arabidopsis plants were used per treatmen. The grades of disease symptoms are described in the Methods section. Different letters indicate a significant difference (*P* < 0.05) based on one-way ANOVA with Tukey multiple comparisons test.

**
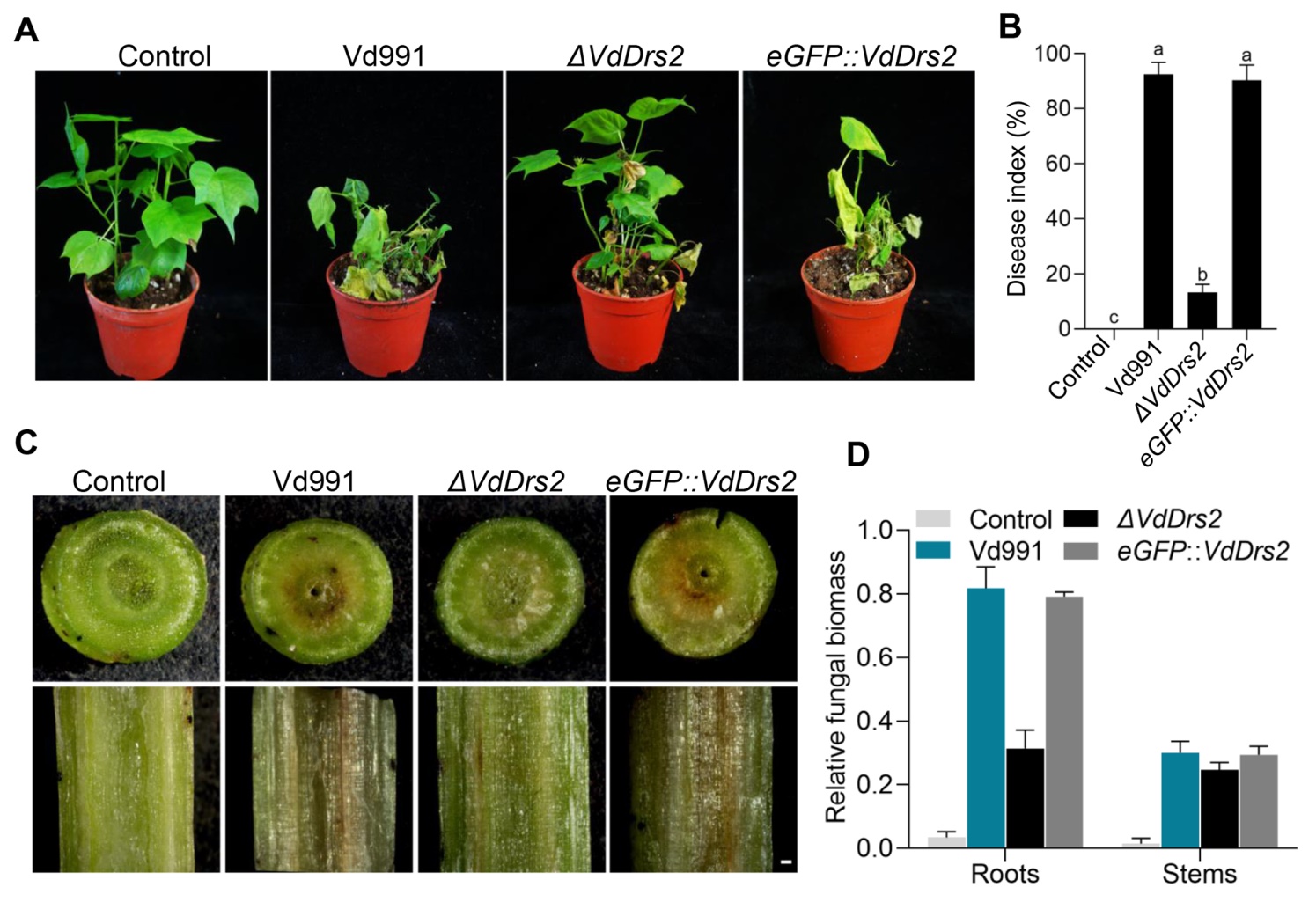
Supplementary Figure 6.** Fusion of eGFP does not impair the function of VdDrs2 in pathogenicity of *V. dahliae*. **(A)** Disease symptoms of cotton plants inoculated with spores of Vd991, Δ*VdDrs2* and *eGFP::VdDrs2* (The strain expressed *eGFP*::*VdDrs2* in Δ*VdDrs2*) by root-dipping inoculation. **(B)** Disease index of infected cotton plants. The grades of disease symptoms are described in the Materials and Methods section. Different letters indicate a significant difference (*P* < 0.05) based on one-way ANOVA with Tukey multiple comparisons test. **(C)** Comparison of vascular discolouration associated with Verticilium wilt in the infected cotton plants inoculated with spores of Vd991, Δ*VdDrs2* and *eGFP*::*VdDrs2* by root-dipping inoculation. Transverse sections (top) and longitudinal sections (bottom) of the infected cotton plant stems. Scale bar represents 0.2 mm. **(D)** Fungal biomass in cotton plants at 14 dpi. The error bars indicate standard deviations from three repeats of the experiment.

**
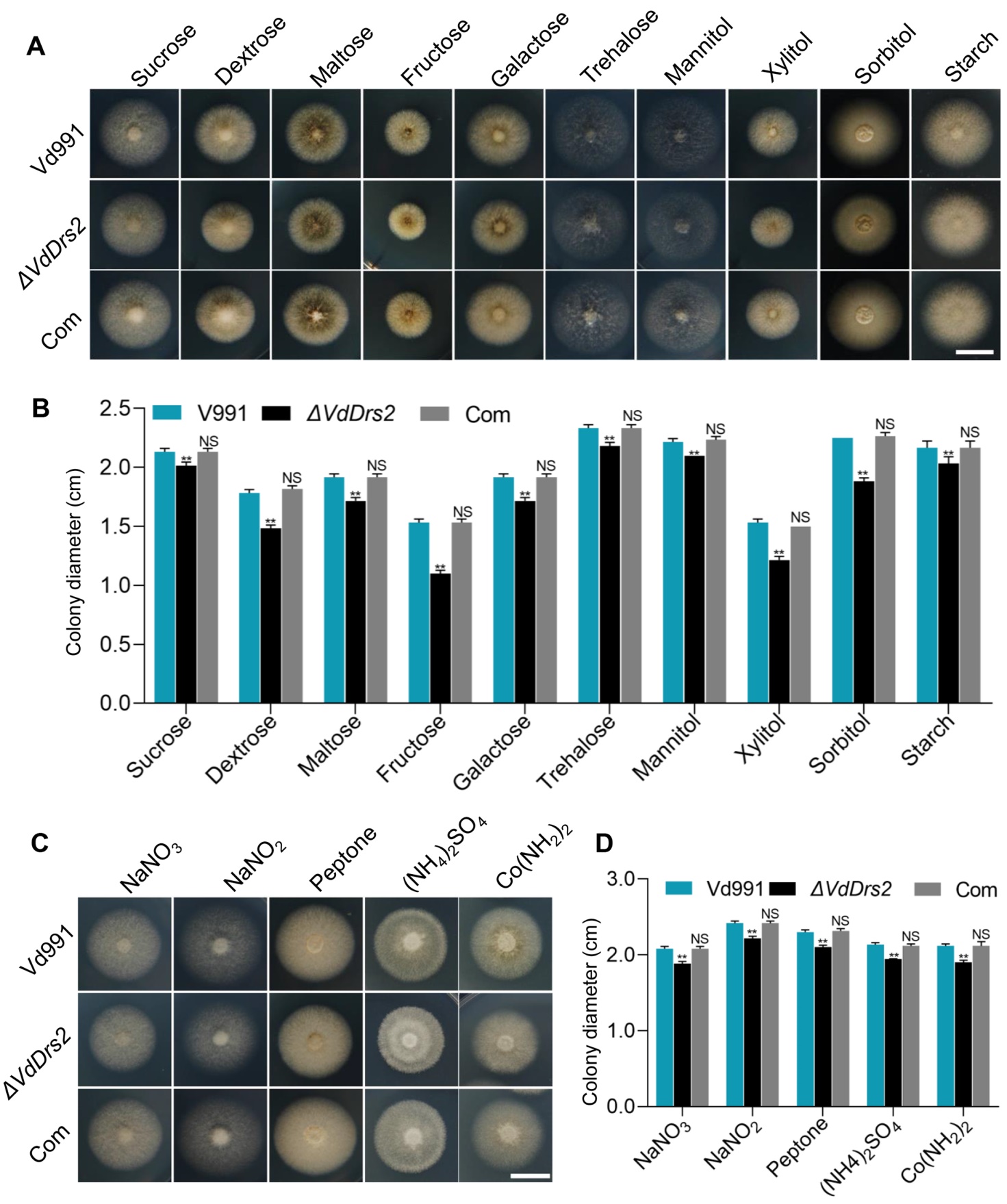
 Supplementary Figure 7.** Colony morphology of the different strains on the media with various carbon and nitrogen source. **(A)** The defect of VdDrs2 decreased hyphae growth of *V. dahliae* on the media with different carbon sources. The concentration of each sugar is 3%. Bar represents 1 cm. **(B)** Colony diameter of Vd991, Δ*VdDrs2* and complemented (Com) strains cultured on media with different carbon source. **(C)** The defect of VdDrs2 decreased the hyphae growth on the media with different nitrogen sources. The concentration of each nitrogen source is 3%. Bar represents 1 cm. **(D)** Colony diameter of Vd991, Δ*VdDrs2* and Com strains on the media with different nitrogen source. Each strain assay was performed thrice. The error bars indicate standard deviations from three repeats of the experiment. Two groups of data were compared using *t* tests in SPSS.**: *P* < 0.01, NS: not significant.

**Supplementary Table 1.** Primers used in this study.

| **Primer name** | **Sequence (5’ to 3’)** | **Restriction enzyme site^a^** | **Remarks/Purpose** |
| --- | --- | --- | --- |
| *VdDrs2-F* | GAATTCatggctggacgacccacggg | *Eco*RI | Cloning of *VdDrs2* gDNA |
| *VdDrs2-R* | GAATTCtcaggttccctgtccttgtg | *Eco*RI | Cloning of *VdDrs2* gDNA |
| *VdDrs2-com-F* | ACGACGGCCAGTGCCAAGCTTatcagatgtggctatcccgg | *Hin*dIII | *VdDrs2* complemented strain construction |
| *VdDrs2-com-R* | AGAACTAGTGATATCAAGCTTtgcaagaactgtatcggtcg | *Hin*dIII | *VdDrs2* complemented strain construction |
| *VdDrs2-LB-F* | tatgacatgattacGAATTCtaagggacctacctatgcac | *Eco*RI | Cloning of 5'-end of *VdDrs2* |
| *VdDrs2-LB-R* | catcttctgtcgacGAATTCagtagtacgagcgtgttgag | *Eco*RI | Cloning of 5'-end of *VdDrs2* |
| *VdDrs2-RB-F* | aggtaatccttcttTCTAGAcgacttgccacaccgtcatc | *Xba*I | Cloning of 3'-end of *VdDrs2* |
| *VdDrs2-RB-R* | gcctgcaggtcgacTCTAGAcgtgttcatgttgagccgtc | *Xba*I | Cloning of 3'-end of *VdDrs2* |
| *VdNeo1-LB-F* | tatgacatgattacGAATTCcagatgcagctgtcttgtca | *Eco*RI | Cloning of 5'-end of *VdNeo1* |
| *VdNeo1-LB-R* | catcttctgtcgacGAATTCaccaggagtacgcactcttg | *Eco*RI | Cloning of 5'-end of *VdNeo1* |
| *VdNeo1-RB-F* | aggtaatccttcttTCTAGActggagtacgcagaaacagg | *Xba*I | Cloning of 3'-end of *VdNeo1* |
| *VdNeo1-RB-R* | gcctgcaggtcgacTCTAGAgctttgagggatggtctgtg | *Xba*I | Cloning of 3'-end of *VdNeo1* |
| *VdP4-4-LB-F* | tatgacatgattacGAATTCtccctgataacatggtctct | *Eco*RI | Cloning of 5'-end of *VdP4-4* |
| *VdP4-4-LB-R* | catcttctgtcgacGAATTCttcagtagaaatgtacacgg | *Eco*RI | Cloning of 5'-end of *VdP4-4* |
| *VdP4-4-RB-F* | aggtaatccttcttTCTAGAaagtcgtcatcgagggcatc | *Xba*I | Cloning of 3'-end of *VdP4-4* |
| *VdP4-4-RB-R* | gcctgcaggtcgacTCTAGAaggatgatgctgtcggcacc | *Xba*I | Cloning of 3'-end of *VdP4-4* |
| *VdDnf1-antisense-F* | gacatcaccatgggAGATCTatccacgataacccgtacct | *Bgl*II | Cloning of 5'-end of *VdDnf1 sense* |
| *VdDnf1-antisense-R* | gatatcgcggccgcAGATCTatcacgctatcggcaccctt | *Bgl*II | Cloning of 5'-end of *VdDnf1 sense* |
| *VdDnf1-antisense-F* | atttatagcccgggGAATTCatcacgctatcggcaccctt | *Eco*RI | Cloning of 3'-end of *VdDnf1 antisense* |
| *VdDnf1-antisense-R* | tggatccgtcatgaGAATTCatccacgataacccgtacct | *Eco*RI | Cloning of 3'-end of *VdDnf1 antisense* |
| *VdDrs2-screen-F* | CACGCCTTGGATCCATGGTG | - | *VdDrs2* mutant/complemented strain screening |
| *VdDrs2-screen-R* | GACCTCGATGAAGACGGCCT | - | *VdDrs2* mutant/complemented strain screening |
| *VdNeo1-screen-F* | CATGGGCAAGAGTGCGTACT | - | *VdNeo1* mutant strain screening |
| *VdNeo1-screen-R* | GTAAACGGAAACACGTCGAG | - | *VdNeo1* mutant strain screening |
| *VdP4-4-screen-F* | CCTTCCGTGTACATTTCTAC | - | *VdP4-4*mutant strain screening |
| *VdP4-4-screen-R* | GCAAGATGCCCTCGATGACG | - | *VdP4-4* mutant strain screening |
| *VdDnf1-RT-F* | cgctgttgtgactatcaatg | - | *VdDnf1RNAi* strain selecting |
| *VdDnf1-RT-R* | acgcttctccgtagatctcg | - | *VdDnf1RNAi* strain selecting |
| *gpdA^mini-^F* | ACGACGGCCAGTGCCAAGCTTtcgacccatccggtgctctg | *Hin*dIII | Cloning of *mRFP*:: *PH^OSBP^* cassette |
| *PH-R* | AGAACTAGTGATATCAAGCTTactagtcaatagtggtgaaa | *Hin*dIII | Cloning of *mRFP*:: *PH^OSBP^* cassette |
| *eGFP-F* | CACCCTTTTAATCAATAACAGCGGCCGCGTCGCCACCatggtgagcaag | *Not*I | Cloning of *eGFP*:: *VdDrs2* cassette |
| *eGFP-R* | ACCAGAACCACCACCAGAACCACCggacttgtacagctcgtcca | - | Cloning of *eGFP*:: *VdDrs2* cassette |
| *VdDrs2-G-F* | ggtggttctggtggtggttctggtatggctggacgacccacggg | - | Cloning of *eGFP*:: *VdDrs2* cassette |
| *VdDrs2-G-R* | CAGTAACGTTAAGTGGATCCtcaggttccctgtccttgtg | *Bam*HI | Cloning of *eGFP*:: *VdDrs2* cassette |
| *eGFP-F*(*Not*I) | CACCCTTTTAATCAATAACAGCGGCCGCGTCGCCACCatggtgagcaa | *Not*I | Cloning of *eGFP* cassette |
| *eGFP-R*(*Bam*HI) | CAGTAACGTTAAGTGGATCCtcaggacttgtacagctcgtcca | *Bam*HI | Cloning of *eGFP* cassette |
| *qVdGAPDH-F* | cgagtccactggtgtcttca | - | Internal reference in RT-qPCR analysis |
| *qVdGAPDH-R* | ccctcaacgatggtgaactt | - | Internal reference in RT-qPCR analysis |
| *qVdβ-Tublin-F* | ATGAAGGAGGTCGAGGACCA |  | Internal reference in RT-qPCR analysis |
| *qVdβ-Tublin-R* | GGTGGAGTTACCGACGAAGG |  | Internal reference in RT-qPCR analysis |
| *ITS1-F* | aaagttttaatggttcgctaaga | - | Detecting of fungal biomass |
| *ST-VE1-R* | cttggtcatttagaggaagtaa | - | Detecting of fungal biomass |
| *GhUBQ7-F* | gaaggcattccacctgaccaac | - | Internal reference in RT-qPCR analysis |
| *GhUBQ7-R* | cttgaccttcttcttcttgtgcttg | - | Internal reference in RT-qPCR analysis |

**^a^ Underlined sequences are restriction enzyme site.**
