# Supplementary material for: Loss of function of VdDrs2, a P4-ATPase, impairs the toxin secretion and microsclerotia formation, and decreases the pathogenicity of Verticillium dahliae
Source: Front Plant Sci. 2022 Aug 22;13:944364. doi: 10.3389/fpls.2022.944364 (PMC9443849; doi:10.3389/fpls.2022.944364)
Supplement: Supplementary file 2 [file Table_1.DOCX]

>BbP4-1

MAGRPPGGPRAGQSHTHDDLLLDLDNEQPVYSQGQRSTLNDDDLLRNYNQPHDSPGRPSVSYDDFVGATGMHESPGRQPGAGTGPHPSSRLDAAGPYMTRQYSQTSELGNYQRYADDLDDYPSDRASYYQNDGASTIGGPANAARQAARNRNSVLSLGGGFLGRFRNRMSRGQGYSEMDLPLTEGGHDRVNGAPPSQQKQAGGGGGGGFDLGNFKFGFGSRKPDPSTLGPRIIHLNNPPANAENKYVDNHISTAKYNFASFLPKFLFEQFSKVANVFFLFTAALQQIPGLSPTNRYTTIAPLLIVLLISAGKELVEDYRRKQADNALNTSKAQVLRGSSFTQTKWINVAVGDVVRVESEEPFPADLVLLASSEPEGLCYIETANLDGETNLKIKQGLPETSTMVSPSELSRLTGRIKSEQPNSSLYTYEATLTMQSGGGERELALNPEQLLLRGATLRNTPWIHGVVVFTGHETKLMRNATATPIKRTKVERQVNSLVLILVGMLLVLSACCTVGDLVTRQVSGNNYGYLYLDRINGVGIALKTFFKDMVTYWVLFSALVPISLFVTVELVKYWHAILINDDLDMYYDKTDTPATCRTSSLVEELGMVEYVFSDKTGTLTCNMMEFKQISIGGIMYSDNVPEDRRATGSDDMEGIHDFKQLRSNLAERHSTAEAIDHFLALLATCHTVIPEVDEKGRIKYQAASPDEGALVEGAKTLGYTFFARKPKAVIIEVGGQELEYELLAVCEFNSSRKRMSTIYRCPDGKIRCYCKGADTVILERLHDQNSHVDVTLRHLEEYASEGLRTLCLAMREIPEQEFQEWHRIFEAAATTVGGNRADELDKAAEIIEHDLTLLGATAIEDRLQDGVPETIHTLQEANIKVWVLTGDRQETAINIGMSCKLLSEDMMLLIVNEETAEGTRDNVQKKLDAIRTQGDGTIEMETLALVIDGKSLTYALEKDMEQLFLKLAIMCKAVICCRVSPLQKALVVKLVKKYQKGSILLAIGDGANDVSMIQAAHIGVGISGVEGLQAARSADVAIAQ

FRYLRKLLLVHGAWSYQRISKTILFSFYKNIALYLTQFWYAFQNVFSGQVIYESWTLSFYNVFYTVFPPLAIGILDQFISARLLDRYPQLYTMGQQNLSFKIKVFWQWIANAVYHSIVLYVFSELIWYDDLIQGDGKTAGHWVWGTALYGAVLLTVLGKAALVTNNWTKYHVMAIPGSMAVWYIFIAAYGTVAPMIPISVEYHGVVPRLYTSPIFWLQTIALAGLCLLRDFAWKYAKRMYRPQTYHHIQEIQKYNIQDYRPRMEQFQKAIRKVRQVQRMRKQRGYAFSQADESQTRVLQAYDTTKNRGRYGEMASSRPQ

>BbDnf1

MPDSLTPEAAAADGADLDKVQTQRARWATRRMTIKSGSTKRRSLLNRHNRNKSSASEKSSAGDDGHGPMSIGAGDEEDDDAEASEASDEDPENRTLFFNLPLPDDMLDDEGHPTTSYARNKIRTAKYTPLSFIPKNIWFQFHNVANIFFLFVIILVIFPIFGSVNPGLSAVPLIVIICLTAIKDAIEDYRRTITDIELNNAPVHRLMNWNNVNVEVGDVSTWRKTKKATSRFFGGIWYAIQSLWSKQARQKRIERKAKAKGQDEEEPRPSIETQRTRMSIRQSIASNFGHRESTHEDIQMTPVPSPSPAGHIKIQEPDKHDQARLAALQDMKTDIINFRHAPTNARFKKDAWKNIVVGDFVRIYNDDELPADVIILSTSDPDGACYVETKNLDGETNLKVRQALRCGRALKHARDCERAEFVVESEAPQSNLYKFNGAIKWKQNIPGYEDDEPEDMTEAITIDNLLLRGCNLRNTEWILGVVVYTGHDTKIMMNTGMTPSKRARIARDMNFNVVCNFGILFVMCLVSAIINGAAWARTDTSKNFFDFGSIGGSPPVTGFITFWAAIINFQNLVPISLYITLEIVRTLQAIFIFSDVEMYYEPIDQPCVPKTWNISDDVGQIEYIFSDKTGTLTQNVMEFKKATINGQPYGEAYTEAQAGMQKRAGIDVSAESDRIHAEIAEAKARSIAGLRKIYNNPYFYDDALTFVAPDFVADLDGESGPGQKEANETFMLALALCHSVIAEKAPGDSPRMLFKAQSPDEEALVATARDMGFTVLGSSCDGIDVNVMGEDRHYPILNTIEFNSTRKRMSSIVKMPDGRIVIFCKGADSVIYSRLKKGEQRELRQETAEHLEMFAREGLRTLCIAMKELTEEEYRAWKKEHDVAASALENREEKLEAAAELIEQDFLLLGGTAIEDRLQIGVPDTIELLGQAGIKLWVLTGDKVETAINIGFSCNLLNTDMELIHLKVDEEAGDDVSDDMLLDELERSLDENLGQFGITGSDEDLRAAKKNHEPPGPTHGLVIDGFALRWALHDRLKQKFLLLCKQCRSVLCCRVSPAQKASVVAMVKNGLDVMTLSIGDGANDVAMIQEADVGVGIAGLEGRQAAMSSDYAIGQFRFLQRLVLVHGRWSYRRLAESISNFFYKNMVWVFGLLWFQIYCEFDITYLFEYSYIIMFNLFFTSVPVGVLGVLDQDVSDKVSLAVPELYRTGIERLEWTQRKFWLYMFDGVYQSVMAFYVPYLIFFNSRPVTFNGLAVDDRYRLGAYVAHPAVVTINAYIMINSYRWDWLMLLIIAISDLFVFFWTGVYTSFTSSATFYKAGAEIYGEASFWACFFIVPVLCLSPRFSIKAMQKVFRPYDVDIVREQVFLGKFDYLNEAKEKDDGKTDSRSQGSHGSSKRSSRKSKHMQYASVDEDLRPIYPPSTVTRDTYNGAHSQQGSADSTHMPRHSVENPLHRLSTDRPRPSYDRMRASMDRTRASFEASNDFTSAARLSRIESSHSRGNSHSHSGMIRSRLRALSSLSKKSDA

>BbNeo1p

mtsspsyrhteppdspthdsdsdldldlqeldpistaptrgskpeltdtsqkssrialrnlrmgglrragkrhrtygelghdrdavdedddsqrllgdrddaqgsedgapllggrsrdnqgrrmslssvlrvpsfmsgtkkgpddeelpeeddpsssrivpvgsvqpvryptnmvsnakytpwsflpvtlynefsfffnmyfllvalsqiipalrigylttyiaplafvlcitmgkeawddidrrrrdneanseqytvlrftdptlesssrpkkllksetmkrgskkgraermeraglsdiqedpesepakpsstivevsrkskdlavgdvlrltkgqrvpadvailqclnsetldkseldeesesadlvalndelesvskgkqpaterrnsaatkvtvgetfirtdqldgetdwklrlaspltqniapeelvrlrvnagkpdkkvnefigtvellpsreeatqqqatlsqeegsattaalsidntawantviasqgstlavimytgphtrsalstspsrsktglleyeinsltkilcaltlalsivlvafegfenrpgkpwyiqimrflvlfstivpislrvnldmgktaygrfiqrdpgipgavvrtstipedlgrieyllsdktgtltqndmemkkihigtvsyaneamdevssyvrqgfyiqpttdpatqsmlitpsstyvntvavgatrtrreigtrvrdvvlalalchnvtpttdeedgkevtsyqasspdeiaivkwaeavglklasrdrksmtlaatttgrpvvrvsildvfpftsegkrmgiivhfqddlkkpnrdlstgdiwfyqkgadtvmssivvandwldeetanmareglrtlvvgrkrlsyqqyqefssryheaslaiqnrdagmqsvvaqylesdlellgvtgvedklqrdikpslellrnagikiwmltgdkvetarcvavssklvargqyiytvaklkrkdnaqehldflrnktdacllidgeslalllthfriefisvavrlptvvacrcsptqkaeiatlikeytkkrvccigdggndvsmiqaadvgvgivgkegrqaslaadfsieqfchlvkllvwhgrnsykrsaklaqfvihrgliiavcqtmysialkfepeglyidwlmvgyatvytaapvlslvldkdvdenlanlypelykelttgrslsyrtffvwvlvsiyqggmiqglsqiltgvenkkmtavsytvlilnellmvaieittwhpimiisilgtflmyigsmpflggyfdlefiitwgfvwrvlailsislvppyaaklirrtmkppsyrkvqnr

>BbP4-4

mhpnridaalhpaeyvssasdaaavdaavniqrnrssaaprmtaasrsrtdpglalgrdnaetarapiserhelqtihskapfaiktdsvddaatsagasaaaansipytkpsfrdrlarlqperltsnkrrrqmhglvwkesvkdwvatayqrviiegllrqkplppshdgrhipmnyphdmidersgrhycsnfirssrytvydfipkqlffqfsklgnfyflvmgilqtipglstvgrwttigpllafvafsmakeglddyrryqldksenrsstlvlsrndgrkrkathaktalariknrnekadvtveetalgdleqtrpssdwieldwqqvrvgdivrlqrdqnvpadmvllhatgpnsvayietmaldgetnlkakqacpplahccntidglrqtqativsedpnidlysydgrvmidgemlpltlnnvvyrgstlrntseaigliinsgeeckirmnahknvrtkkpamqstvnlmilfqifivvmlavgftigyylweedvenrsfylyrngfydasvpfreiffgflimfntliplslyisleivklgqllllhdadmydpvtdtpmvantttilenlgqishvfsdktgtltenlmrfrklsvagisvlhdmdilrdeklkqvkldsrkvskkrgkqlanviasadpvgrtstgsfthwkssvrpndepdmkteelleyirskpntsfsrkakhfllcialchtclpeckddgsiefqaaspdelalveaaqdlgymlidrpaqtiklqtidaegamvvdsyqvldviefssqrkrmsiivrmpdnticvickgadnvitsrlklshlaeqkardigrrasmrktfeknkalqrksmqlsvrstprtslamrnrestdirdslrqsigrrsvdgarlgeglsswlqrrhieettsprhsvellksnrqslsrvapfdaidrrvdesvassdaatfekcfqhvddfateglrtllygyryidedtytkwkaqyreaetslvdrqqrieeagdviehkfdlagataiedklqdgvpetidklsranikvwmltgdkretainiahsahvckpfselyildanavatltdqltmtltdvsrgmlphtvlvvdgqtlteidadldlsvlfydlvvrvdsiiccraspsqkaelvkairryvpnsmtlaigdgandigmiqashvgigisgreglqaarisdysiaqfrflqklllvhgrwnylrtgkyvlatfwkeiffylmqahfqrftgytgtslfqstsltlfntlfsslavilpgiferdlraetllavpelytfgqrnkafnyrlyigwmlmgiassfivfyiswavyssalftedtslyamgticftvgvvfintklfilelhsktiitfagyvvtvggwfiwlliltkaispamnvylvydtfirnfgarlewwimlllaltgplmlelviqsvrrvywptdtdlmqrmekdadtqqmqalfdqdpeshrhddhdshddeeeasadhqrgrnatklsqeidrrpgharfsqsdvprrvpamrasldesrrpqftpaavkresslnrlptkleqirdd

>BbP4-5

mavknstletdslpdnaavphtrasvlffskvqhlvksayrksivkpllrrkslapsdggrrvplsikhaeplvdtrhgfsyisndirtsrytvfdfipkqlffqfsrvgnfyflcvgipqmipnlsttgtfttilpllffvlltivkegyddyrrsrldrienasftttlgredhytgkvkpptwvqrwnpfhvttdaqppalpedeydgvrwvpirwsnvkvgdvvrlnrddavpadivllwtsdendlayidtmaldgetnlkskqlssalggcgtiegianckanffvedpnpqlfnfegsvsadgksvplslneviyrgsvvrntaaivgivintgeeckirmnsnqhpsakkpalekivncvvvtlatyvvvlsvgvcmgyvhwqrsyerqaqylnnatiplyqiiiafiimfnnvvplalyisleivkigqllllnsdigmydeetntparcntntilenlgqvgyifsdktgtltdnimkfrkisvagtvwlhemdlvddgentatslgdssrepslykpdpvptsplshmssplyprmsigarpsisrrrssshwrstgrpdhiqpevntadllhyiqlrphsafarkakeyilamalchtclpeytngniefqasspdelalvraaqemgylvaqrtakkvtvemtqangqldrqtydildtvefssnrkrmsivlrrpdgritvickgadsviiprlkqsslamqkatearasadiehemqrrseqhqprnsfggrpsltvrynsnihrpqqsmprsviyrsksfelrklvrssedgvrasactrgmsmdinrahvrtsvnthhvqlphnlqflddpiihdefeafskcfkhiedfateglrtllfaqkdiseqdyrawkriyddattslvnrqemlesagdlleqafsligasaiedklqkgvpetieklrraniriwmltgdkretainiahsarlvrpgsdiyildiakgalefqlialiedlqigpfdrivvidghtlsviensaelskrfydimlkvdtviccraspaqksllvrtvrdriskfnltrrhgltlaigdgandlamiqashvgigisgreglqaarvadysiaqfrflqrlllvhgrwnyvrttkfilctfwkemffylpqaiyqryngytgtslyeatsltvfntlftslcticmgiweqdlraetllaipelyvygqrdmglnvwkyarwmllaaiegvitwygvwagygwihprardeglyalgtltfttgvmwinwklfifethykasivmgsffvttigwfawisfldgifaatasgpydvkhsfrykwgpdavwwatlfavlgtlwllemtmkvikrhlilcgvwkwppwkksslsenveewdvglwqeleqdatvqaklkvmarehigqevdgelveedigeyperilveetveillylrryylfkgp

>FgDnfA

mttttsadghlaapddsqhlsatqrsrwatqrksvnssnnkrnsildrmghkktgsneknppsdgsdpagddgqspeanpdnededeehenrtlyfnqplptelldengapsqtytrnkirtakytpisfvpknlwfqfhnvanifflflvilvifpifggvnpglnavplifiiavtaikdaiedyrrtvldielnnapvhrlrnwnnvnvlegdvstwrqfkkanskffgsiwratqsiwskkakeerakrkavsteedgprasvetqrtrqsmrqsiaspftgresfmsarediqmtpvpspspqatpharfempdeqdakratalmqmkpdvinyhhpasgarfqkdtwkslnvgdfvriyndeelpadviilstsdpdgacyvetknldgetnlkvrqavrcgrslkhardceraefvvesegpqpnlykyngaikwkqsvpgylddepedmtepitidnlllrgcnlrntewivgvviytghdtkimmnagitpskrariaremnfnvvcnfgillimcllaaiingvawaktdaslhffdfgsiggkpamsgfitfwaaiilfqnlvpislyitleivrtlqaifiyndvemyyepidqpcipkswnisddvgqieyifsdktgtltqnvmefkkatingqpygeayteaqagmqkrlgidvekegervraeiadakvralaglrnihdnpflhdesltfiapdfvsdlagesgpdqkeaneffmlalalchtvmaekvdgdipqmifkaqspdeealvatardmgftvlgssgeginlnvmgedrhyqilntiefnssrkrmssivrmpdgriilfckgadsiiysrlkrgeqkelrkttaehlemfareglrtlciawkevtehdyrvwkkehdaaasaleereekletvaelieqdlylvggtaiedrlqdgvpdtiallgnagiklwvltgdkvetainigfscnllnndmelihlkvdedesgeitdeaffemaekllddnlqifgitgsdhdlalakknheppapthglvidgftlrwvlndrlkqkflllckqcksvlccrvspaqkaavvamvkngldvmtlsigdgandvamiqeadvgvgiagvegrqaamssdyaiaqfrflsrlvlvhgrwsyrrlaesisnffyknmvwtfsifwyeiycdmdmtylfdytyilmfnlfftsipvaimgvldqdvsdkvslavpqlyrrgierlewtqlkfwlymidgiyqsimvffipyllfmpgtfltgnglgledrlrfgtyvahpavitinmyilintyrwdwlmvlivvisdvfiffwtgvytsftssqyfygtaaqvygeatfwacfflvpviclfprfaikalqkvywpydvdiireqermgnfayldktdetndpptadtksdksksskssrrskkmpkhvaygsvdedlrpiyppstatrattynqhsqngsdstnytahrisldvpmqgrpsidrarpsydrmrasmdrvrpsfeasndftsaarlsriessqsqtgrfhprlrglsltksani

>FgDnfB

magrppgggfntghsnnrddllldldndqpvygggqrsnlndddlmrfheqnqdpppgrtsvsyddfvgardanhppashppgalgvpgsgpgsnpylnrqysqtselgnyqryaddfddypaegdsyyhndggarvdertpglgmnsarnrnsvltmgggfigkvknrlgmgqgysemdlpltqpghdrgdsvggqsqmppqqgqkgrfdmgnfkfgfggskpdpstlgprviylnnppanaankyvdnhistakynvasflpkflyeqfskfanifflftaalqqipnlsptnpyttiapltvvliisagkelvedyrrkqadnalntskarvlrgsnfeetkwinvaigdiirveseepfpadlvllassepeglcyietanldgetnlkikqaipetsamvspnelsrlggrikseqpnsslytyeatltmqmgggekeyalnpeqlllrgatlrntpwvhgvvvftghetklmrnataapikrtkverklnwlvlllvgillilsivctvgdliqrkvegnalsylyldptntagqitqtflkdmvtywvlfsalvpislfvtvemvkywhailinddldmyydkndtpatcrtsslveelgmveyvfsdktgtltcnqmefkqcsiagiqysedvpedrrptmidgvevglfdykalksnlanghetapaidhflsllstchtvipemdekggikyqaaspdegalvagaldlgykftarkpksviidangreleyellavcefnstrkrmstiyrcpdgkircyckgadtvilerlnehnphveitlrhleeyaseglrtlclamreipenefqewykiydtaqmtvggnradevdkaseiiekdffllgataiedrlqdgvpetihtlqqanikvwvltgdrqetainigmsckllsedmmlliineetaaatrdniqkktdairtqgdgtietetlaliidgksltyalekdlekmfldlaimckaviccrvsplqkalvvklvkkyqkesillaigdgandvsmiqaahigigisgeeglqaarsadvaiaqfrflrklllvhgawsyqrvtktilfsfyknialymtqfwytfqnvfsgqviyeswtlsfynvfytvlpplalgildqfisarlldrypqlymmgqqnyffrlkvflewianaiyhsivlyiwgeliwhgdliqgdgkiaghwvwgtalygatlltvlg

kaalvtnnwtkyhviaipgsmaiwyvmtavygivapmagvsmeyhgtipriyespifwlqtvclaimcllrdfvwkyvkrmyrpqtyhhiqeiqkyniqdyrprmeqfqkairkvrqvqrmrkqrgyafsqadesqtrvlqaydttkhrgrygemassrpagr

>FgDnfC1

mapypsdnngrrppqqpgnnddddvardlaalderypptsanfsrprntrrsytdpnndrpprrssaddtnvrsasrsrsrrpddgarrrasgpyelrpipsrtelstdlsgppdygdradvsvdkasvayngnsssrekssvradikkylrqrrekkigpsakptwkertrkqlgelhqkviveailrqkplqplpdgrhvplnpahrnanglideragkpyisnfirssrytvydffpkqliyqfsklgnfyflvvgtiqmipglstvgrwttiaplgvfvafsmakegyddyrryrldrvenrseawvltdraaatekgrirhaekmkkrkekesrngeehmlddietgvtgakkvgsdgdwtsvqwqhvrvgdvirlrrddavpadivllhatgpngiayidtmaldgetnlkskqacpllaercntmeglratqatvisenpnldlysydgrvtvdgetlplsmnnvvyrgstlrntaealgiivntgeeckirmnanknvrakkpamqsvinkmimvqifivlmltmgltigyylwrdrtedfawyirrrgfwdaripfkeiffgfiimfntliplslyisleiiklgqlfllqdddmydpisdtpmvantttilenlgqvsyvfsdktgtltenimrfrklsvagvaclhdmdvqrdqeemrrkieeserpkkgksrmaslsasrsavptitidgqqgdlqrpeptrtmstshwqssvttaqntdmktedlldyiqrkpntafsrrakhfllcialchtclpektdegdinfqaaspdelalveaardlgflvidrpaqaikletrdtdgslhtesyqvldviefsskrkrmsiiirmpdgricvfckgadnvimqrlklsnlaeekakdigrrtsqrrlsrqdqalrrmstqqsispygspygsprnsfgfsraesssnreglrlslgrrstdlkrlsqqlarsprastdmmspisprqslghmpsfenadskideslasnegaifekcfqhvddfaseglrtllyayryidedsyaqwkakyreaetslvdrqerieaagelieqkfelagataiedklqegvpdtidklrranikvwmltgdkretainighsarvckpwsevyvldatlgelketitmtlndvsrgmvahsvvvvdgqtlakideddelsllfydlvvrvdsviccraspsqktnlvssirryvpksmtlaigdgandigmiqashvgigisgreglqaarisdysiaqfrflqkllfvhgrwnymrtgkyvlatfwkeilffivqahyqrytgytgtslyeswsltvfnsaftslpvillgifekdlraetlmkvpelytfgqfnlgfrfsqyfawmimgvagsfiiwyftwcvydksfsdqdtsifamgmvsftvavvfinvkllilevhtktvitfggclvsvagwflwmlalsgikpatigpyivrdafidnfgrtlqwwtivllelitlvfielvvqavrrvyfpsdqdlmqriekdgnvdkvfgdgkdaeegdaeaeervipeagervrerragrashddyqpeflptaeeerenpmeqwrr

>FgDnfC2

msqdrpdvgaplgpepsktdsktntrrrrsdagqsaplanrikgaaadlyqktvvelilrrkhivastdgrhvplkleheapliddrrglpyvsnsirtsrytvwdfipkqlffqfsrvgnfyflcvgvpqmipglsttgsyttilpllffvlltivkegyddyrryrldkvenagfatvlgredkytgkikpvtkwrkwnpfmtnstaqphpapneefnglrwvpvrwseiqvgdiirlcrdepipadlilldsdeenklayietmaldgetnlkckqvahalqgcdtiegiskckaefvvedpnpdlynfdgrvtvnektvpltsseviyrgsivrntsaaiglvintgedckirmnankhpkakkpalervvnkivvslatfvvilsvgvsmgyvrwqksterhswyleqakvpfyqiiiafiimfnnvvplalyisleivkigqlimlngdlqmydeetdtparcntntilenlgqvgyifsdktgtltdnimkfrkisvagtvwlhemdleqkvdeieaikldeesdpgepsayktepvtvvireeqpeashepstplalpspshmsprpsmsrrpsmapsrpsmaysrpsfgerrsssqwrstgrpdhiqpdvttndlieylrlrpnsgfakkakqyilavalchtclpehkengelefqaaspdelalvraaqelgylvinrttqtitlrvtqsdgqeedqkyevldvieftsarkkmsivvrfpdgrvsvickgadsailprlkmsqiakqkanevrksadieremrrrseqqeprnsfggrpsltirrnpgisrdrstsrrpnvdrsksfefgrlsrrsedkprlsiatrgvsidmprgqylhtpvhyqqpvpdhlafledpallddsetftkcfkhlddfateglrtllfaqkfiteheyqawkkvwdeaatslsnrqqrieeagdmieqsfdlvgataiedklqqgvpetierlrkanikiwmltgdkretainiahsaricrpgsdlyildvskggldsqlialqedlqagsvhsvvvidgqtlsavekspelsakffkimlqvdsviccraspaqkallvttvrsrlkkyrgknrrgltlaigdgandlamisashvgigisgkeglqaarvadyaiaqfrflqrmllvhgrwnyvrtskfilytfwkemffylptaqyqrytgysgtslyeatsltvfntlftslcvicmgvweqdlsadtllavpelyvygqrnqglniwkfarwmllgaiegvicwygvwagygwitpaardqglyalgtltftagvlwinwklfmfethyksiivmisffvttigwfawlsfldaayapqpsgpyaisdsfttlfgddavwwatlfivlgliglfemilkcvkrlllmdglwdwppwgksrrgenieewdvelwqeleqdpalrarlkrmardepveeeddvdlaqinideemrgr

>FgDnfD

mppsqqyrpsdppdsptndsdsdldldiqeldpisttpapratkehpptepetsrialrnlrmgglrragkrnrgygdlgrdrdgndehseallgdrnsstpqwsegsgyneddqpllggqpsqgrrsinsdrvssrlrlpsfmssskksdtndaedredddpsssrhvavgstqpvrfpnniisnakytaltflpitlynefsfffnmyfllvalsqaipalrigylltyiaplafvlcitmgkeafddiarrrrdteanseeykvivfqdadltqtpvrqrkllksevlqkhsrkkskgarenlsdileeenveapppssraievsrkskdlkvgdvlkltkgqrvpadvvilkclssevpapapipeepaeeetllafdgeepqgkgkqpaeaapeqeaesgpggetfirtdqldgetdwklrlaspltqnlpteefvrlrvtggkpdrkvneflgtielvesrkdalahhatvgesdashtaalsidntawantviasqattlavimytgpqtrsalstapsrsktglleyeinsltkilcaltlalsiilvaleg

fgntkdnvwyvkimrflvlfstivpislrvnldmgksayswfiqrdpgmpgavvrtstipedlgrieyllsdktgtltqnememkkihvgtvsyaneamdevsayvkqgfyvppstdhvsqnmlitpsstysssanigttrtrreigtrvrdvvlalalchnvtptvdiedgkevtgyqasspdeiaivkwtesvglklvyrdrksmvleyanskrpvvrvrildvfpftsegkrmgiivhfhedasvknpslssgeiwffqkgadtvmssivasndwldeetanmareglrtlvvgrkklsygqyqefaaryqtaslsisgrdagmqrvvshyleneleligvtgvedklqkdvkpslellrnagikiwmltgdkvetarcvavssklvargqyiytvskltkkdnaqehldflrsktdacllvdgdslalflthfrlefisiavqlptvvacrcspnqkaevaklireytkkrvccigdggndvsmiqaadvgvgivgkegrqaslaadfsieqfchlvkllvwhgrnsykrsaklaqfvihrgliiavcqtmysiaikfepeglykdwllvgyatcytaapvlslvldkdvdedlanlypelykelttgrslsyrtffvwvlvsiyqggmiqglsqiltevdgpkmvavsytvlvlnellmvaieittwhpimiisiigtfllyigsipflggyfdleflitwgfvwrvlaigaislvppyagklirrtmkppsyrkvqnr

>MgPde1

MAGQQPPGRPGAPRSNNDLLLDLENDQPVYGGGQRSNVNDDDLLRFHNQDQAAGRPSVSYDDFVGGGGGRDQYTSPPSSSRQTPQDRPETATSQRGPYMQPTARQYSQTSELGNYQRYADDFDDYPEDGQSYYQHGGAPRADDPGNQSRDNARKRNSVLSMGGGLMGRAKNMFGMGGSGYSEMDLPLTEPGGQQRGGAPGGAATGDASHQQPSQKKSGGKFDMGNFKFGFGRGKPDPSTLGPRIIHLNNPPANAANKYVDNHVSTAKYNFATFLPKFLYEQFSKFANIFFLFTAALQQIPRLSPTNQYTTIGPLIVVLMVSAGKEMVEDYRRKMADKALNMSKARVLRGSSFEETKWINIAVGDIVRVESEEPFPADLVLLASSEPEGLCYIETANLDGETNLKIKQAIPETSHLVSSSELSRLGGRIRSEQPNSSLYTYEATLTMQAGGGEKELPLNPEQLLLRGATLRNTPWLYGVVVFTGHETKLMRNATAAPIKRTKVERQLNIAVLGLVAILLVLSVVCTVGDLVTRSVFGGSISYIMLDNATDALEIFKVFLRDMVTYWVLFSALVPISLFVTLEVVKYWHGILINDDLDMYHDKTDTPANCRTSSLVEELGMVEYVFSDKTGTLTCNMMEFKQSSIAGIMYGEDIPEDRRATVQDGVEIGIHDFKQLAQNLKTHKTAPAIEHFLALLATCHTVIPERDEKSDKIKYQAASPDEGALVEGAAQLGYKFVARKPRAVIIEVEGQEFEYELLAVCEFNSTRKRMSTIYRCPDGKIRVYCKGADTVILERLNESNPHVEVTLQHLEEYASEGLRTLCLAMREVPDHEFTEWMAVYEKAQTTVSGNRAEELDKAAELIEHSFYLLGATAIEDRLQDGVPETIHTLQNAGVKVWVLTGDRQETAINIGMSCKLLSEDMMLLIVNEETAAATRDNIQKKLEAIRTQGDGTIEMETLALVIDGKSLTYALEPELDKMFLDLAIMCKAVICCRVSPLQKALVVKLVKKYQKESILLAIGDGANDVAMIQAAHIGIGISGMEGLQAARSADVSIAQFRFLRKLLLVHGAWSYQRVSKAILFSFYKNITLYMTQFWYTFQNVFSGQIIYESWTLSFYNVLFTVLPPLALGILDQYVSAGLLDKYPQLYGIGQQNKFFKFKNFAQWIANAMYHSLLLYLGAVVFWYHDLIQADGLIAGKWVWGTALYGAVLLTVLGKAALITNNWTKYHVLSIPGSFVIWVVFIVFYASVFPRFNISIEYDGLVPRLFPSAVFWVQLVVLPVLCLLRDVAWKYAKRMYRPEAYHHIQEIQKYNIQDYRPRMDQFQKAIRKVRQVQRMRKQRGYAFSQADESQTRVLQAYDTTQNRGRYGEMASSKGR

>MgAPT2

MESSNAAETSTLTPVATTQRARWATRKMTIKSSGLKRLSLLGRAQNRGSSTEKKRASMGKGGESLQQSSEGDNEDQDQGEHDDQPEGPGPRTLYFNLPLPPEKLNDKGELVDTYPRNKIRTAKYTPLSFIPKNLWLQFHNIANIYFLFLVILAFFPIFGSVNPGLGAVPLIFIVTVTAIKDAIEDSRRTQSDIELNDSTVHRLCGWNNINVKEDNISLWRRFKKGNTKVFGGLWRFMETLWSKEARVKRREKAAQVADTPRISIDTRRTGRPESVISPHTHRTSFISAREDIPMTPVTSPLPRAEGQQNLEVVPQQEGLASGRPSSNFSFRGDSAELAHIKTDLINRTIPAEGKARFRRDKWKNLQVGDFVRIYSDDELPADIIILATSDPEGACYIETKNLDGETNLKFRQALKCGRNMKNSRDCERAQFIVESEPPQPNLYKYNGAIKWNQPIEGDASGSWREMTEPITIDNTLLRGCNLRNTDWVLGVVLFTGHHTKIMMNSGITPSKRPRIARELNYNVLYNFFILFGMCLLSAIVNGFAFGTSDNSIALFEYGSIGPTPAMNGFITFWAAIILFQNLVPISLFISLELVRLLQAFFIYSDVDMYYEPIDQPCIPKSWNISDDLGQIEYIFSDKTGTLTQNVMEFKKATINGQPYGEAYTEALAGLHRRMGIDVEKEAAEARVQIAADKEKALAALRKIHDNPYLHDDDLQFIAPDFVEDLMGANGQEQQQACERFMLALALCHTVIPERQPGEKATMLYKAQSPDEAALVSTARDMGFTVLSSNSDGVRLNVMGEEKYYPILNTIEFNSSRKRMSAIVRMTDGQTVLFCKGADSIIYSRLKKGEQKQLRTDTAQHLEMFAREGLRTLCIAERVLGEQEYQAWSKEYAVAAAAVENREDKMEAIADQIEQDLTLLGGTAIEDRLQDGVPQTIAVLAEAGIKLWVLTGDKVETAINIGFSCNLLNNDMELLNLKVDEDETGLTTREQFMAKLEAELDKYLKIFNLTGSDEDLAAARKIHEAPQATHAVVIDGFTLRWVLEDTLKQKFLLLCKQCKSVLCCRVSPAQKAAVCAMVKNGLDVMTLSIGDGANDVAMIQEADVGVGIAGVEGRQAVMSSDYAIAQFSFLQRLVLVHGRWSYRRLAESISNFFYKNLVWAMPLFLFQIYCDFDMTYLFDYTYILMFNLLFTSVPVILMGVLDQDVSDTVSLAVPQLYRRGIERLEWTQTKFWLYMLDGTYQGVMSFFIPYLVVVGSPFVTTNGLDVTDRVRFGAYIAHPAVVTINLYILINSYQWDWLIVLVVVLSDLFVFFWTGVFTSNTYSQWFYEAAPQIYAQPSFWAVFIITPVMCMFPRFSIKALQKVYWPYDVDIIREQVQQGKFDRLQPAPVGQTLPGGRSTAGSSPSLAKGNPGTFSGQDDERPIYPPSVATHTRTQTGSDGTNYTGHRMSMEQQQLEHQPLDQLTPMEHIPTRPSIDRARPSYDRVRASMDRVRPSYEASNDFTSAARLSRVESSRSRGGPPPSSHNLFAGRLRGLSIISAKSRHGPDSRGGQ

>MgAPT3

MPNQQASHHHPSKPPDSPVDDSDSDLEIDLQELDPQSNSSQRPLRGSAESYAREAAEHRSSPRIPLRNLRMGGLRRSRDRGQYGELGHRRDGSSDQSAGLLTPGDRDASSSRFEDSDRHGDDDAPLLPDSEQGRRRPGGHRRLKSITDALRLPSFMSSSSNVADQRQPLPEEDEGQEDHDPSGSRLVAVGSAQSARFPPNAVSNAKYTAWSFLPITLYNEFSFFFNMYFLLVALSQAIPALRIGYISTYIAPLAFVLMITLGKEAHDDIARRRRDSEANSEPYTTIQFDDPGATVKNGRSRKKLKSAGSRKAQLRLSERDRLSDIQEEEELAEGDGAGKGASSHIREVLKASKDLKVGDVLRLTKGQRVPADVVILQCLASEVTASTPVASKLAEPELLTLDDASGNGGASQPRDDGASDGDGSQAGAGGETFIRTDQLDGETDWKLRIASPLSQNLATEELVRFRVTAGKPDKKVNEFVGTLELLPSRQDAMSPGARLPNGDELANTAPLTIDNTAWANTVIASRAVTLAVIVYTGPQTRSALSTSPSRSKTGLLEYEINSLTKILCALTLALSIILVAFQGFTNTEGNVWYIKIMRFLVLFSTIVPISLRVNLDLGKSVYSWFIQRDPGMPGAVVRTSMIPEDLGRIEYLLSDKTGTLTQNEMEMKKIHIGTVSYANEAMDEVSSYIKQGFHVQPTLSDTSESIQLVTPSSTFTNAAASGTTRTRREIGARVRDVVLALALCHNVTPTTDEEDGKTVTSYQASSPDEIAIVKWTEAVGLRLTYRDRQSMTLSFSETGKTVVHVRILDIFPFTSEGKRMGVIVQFFERPNEIGSGETWFYQKGADTVMSSIVAANDWLDEETANMAREGLRTLVVGRKRLSAEQYREFSSRYQEASLAINGRDAGMQKVVSHYLERDLELLGVTGVEDKLQKDVKPSLELLRNAGIKIWMLTGDKVETARCVAVSSKLVARGQYIYTLAKMKRTDSAQDHLDFLRNKTDACLLIDGESLALLLQYHRLDFVSVAVRLPTVVACRCSPTQKAEVARLIKDYTRKRVCCIGDGGNDVSMIQAADVGVGIVGKEGRQASLAADFSIEQFHHLTKLLVWHGRNSYKRSAKLAQFVIHRGLIIAVCQTMYSIAMHFEPDGLYKDWLLVGYATMYTAFPVLSLVLDKDVDENLANLYPELYKELTSGRSLSYRTFFVWVLVSIYQGGIIQGLSQLLTEVDGPKMVAVSFTVLVLNELLMVAIEITTWHPVMIVSIVGTFLVYMGSIPFLSGYFDLKFMITLDFLWRVLAILAISLIPPYVGKLIRRTMKPPSYRKVQGI

>MgAPT4

mapplqaiggdtrvpaqrqtrrrhlriadaihgtsdsasaiwrryvsanlervaaaipsfhrsippsrdgrqvplsarwrdgvelrdertgkpyisnfirssrytiwdflprqlvfqfmkianfyfliiailqlipglspvasyttgaplivfvafsmakegyddyrrykldlvenrssawvldpngvvksrlerskkgkigvkrpskrertddeagqdfgdgtelrqigegdqaggskqdrhtdhhstarqniwshvqwqdirvgdvvrlqrdeqvpadlvllsasgtngiayidtmaldgetnlkskqacpllakrcgtvagichcdatviaedpnldiysfdgsvrvgdktmpltrdqvvfrgstmrntreayglvinsgeeckirmnahksvrakhpamqavtnrivlllivvlvmlavglkigndefeaefgptnwymfgvrlsiseqvfgfiillntliplslyisleaikigqlymlqdiemydaesntpivantttilenlgqvsyifsdktgtltenvmrfqklslggyvwnhdmgirdeapetelqtlglmqhlqynpetplseralhfilcmalcntclpettdkgeieyqaaspdelalinaarelgyvmidrdsetiklqrpvsgsnddvdigtytildvieftsdrkrmsvilgtpegrilllskgadsallprlrlkqlvdrvaaavnrrtnrrrsmrmdsarkmsgpvspssigewsdfglhddhtssevamlsdadifeacfrhiedfaseglrtllfsyryieeadyvawkreyheaetslvdrqaridsvaekielelelagitaiedklqdgvpetidklrranikvwmltgdkretainighsakicqpfsdifildasaegphidetmdgvlrevkegaiehsvvvidgltltevenskaskdlfydlltrvdaviccraspaqkavvvqcirdkvpgsltlaigdgandvamiqashvgigisgkeglqaarisdysiaqfrflqrlllvhgrwnyvrtskyilgtfwkeivfylnqalfarynaytgtslfesasltvfnalftllavvlmgvfeqdlsaetllafpelyvlgqqdkglnfvkyfgwmvlgaaealvifftvfaayspiysdddttifpignavftavvifinikmllvemhhktwivlggflititgwfvwnlflalvyhrsigpytvrgsfihgfgrvpkwwlaviaalmaalmlelivksirriyfpsdttimqeieaeqrrkqsrankrhqaeraqeegrl

>MgAPT5

mvsivshkakapskeslatdiepgnpptaprqvgarqrasdwfakvsqeglfhkplppskdgrhvpirfagsqdgdkathliderrghgylsnairtsrytlydffpkqvwfqfsrlsnfyflcvgipqtipgisttgnfttilpllffvlltivkegyddykrhrldkvenarcatvlrrasgadevletqrqsgwplphflgkhfqqkswedrrepagegwewrtvqwrdievgdvlrlsrnedvpadlilvqaenedhiayvetmaldgetnlksksvpanltklasisdivqsgleivaedpnpdlhnfdgrltiagdtapltinevlyrgctlrntpaavgvvintgeeckirmnanrhpkakkpalekttnrivltlvayvfsctagcsigyllwqrstensswylrgagvavqeivvgyaihfnniiplslyvtleltklfqmlmlngdiemyheasdtpakcntntilenlgqvgyvfsdktgtltenvmqfrkmsiagiswlhdaglqkpdglspvstsvqvisptssnremavptvvmshqperrsfvipedseipydvprgrpqetemfprrsglsatsprrsslqwrssgrpdleqpglssadlmeyirhrpnsafaraatdyllalalchtclpeysddgidyqsaspdelalvraaqelgyqvvqrsshsvtlrlgstnggedtrevvyeildvvefsskrkrmsiiircpdgrillickgadsvilprlrqanlalqkvqevrqsvevekealrhseareprnslggrpsldlrrhggyadaglsgpfakrpsmsdrsksfeahsrarrsadlvrpslqlrtmsfdlkrsrndpsgsallsapaltqlpakfevledpsvnddgvvftrcfkhlddfaseglrtlvyaqkflsesdyaswkklysdattslvdrqerietvselleqnlelvgataiedklqkgvpetidklrranikvwmltgdkretainiahsarickpysdvhildsskgniegqlagiieelreqknsnglvpsfqnnhnvlvvdghtlgemepsvhlselfyslapvvdsviccraspaqkallvravrqkiiqstpsgksatrgdtlltlsigdggnd

lamlaeahvgvgisgreglqaarvadysiaqfrflarlllvhgrwnysrtarfvlatfwkemyfytgtiayqyfvgytgtslyemwsltalnllftslctispalweqdlsattllavpelytfgqrnlgldvtkylswmlagvaeglitffscwasvgvfgltgdiglyaignlafsiammwtnwklliiethtknivilgsflitvagwwawqaflsgsyspspspyavrggltegfgrdlswwtclivvlaalivfemgykavkrqlvvggalrkrqrkakrvlgrilhrvlcglfvstgvaaaagiktgdaqaddlrrqealeneedldlelwqeleqdpevrarleglcwvedeeenvgdddednaeevqvgrtga

>NcDnfA

mtprpadedeggaeptpitatqrsrwatrkltvkssglkrlslknrnnhgnavgiekkrasghsgksdstagqsdtppptagghagkddadeqhseprtifcglpladefkddeghptqqyprnkirtakytplsfvpknlwfqfhnianifflfvvilvifpifggvnpglnavpliviicvtaikdavedyrrtvldnvlnnapvhklhgipnvnveadnvslwrrfkkansrffgalwhkteglwkkeddnpilknhtashdepdprmsmesrtsrarrslnvvrdevemtpvpsplphqpnemdfpgegssrqvdnkyalqdikgdlvnrnlpisgkarfakdawkglvvgdfvriynddeipadiiilatsdpdgacyvetknldgetnlkvrsalrcgrgmkhardceraqfiieseppqpnlykyngavrwlqelpndedgdpipmsepisidnmllrgcnlrntewalgvvvftghdtkimmnagvtpskrariarelninvvynftillimcliaaiangvawaktdassywfewgsiggtsgltgfitfwaavivfqnlvpislyisleivrtlqayfiysdinmyyepidapcipkswnisddvgqieyifsdktgtltqnvmefkkatingqpygeafteaqigmskrsgggdieseiarikdeieqakartlhglreihnnpylhdedltfvapdfvedlagkngpeqqkanehfmlalalchtvvaekqpgdspkmifkaqspdeaalvatardmgftvlgmsdggvnvnvmgkdmhypvlniiefnssrkrmsaivrmpdgkiklfckgadsiiyarlkrgeqkelrretaehlemfaveglrtlciaekelteqeyyewkkehdvaatalenreekleevadkieqdltllggtaiedrlqdgvpdaiellgnagiklwvltgdkvetainigfscnllnndmdlvrlqvsedeagvqqeaeylrlaeeeldrglakfgmtgsdeelrqakkdheppapthglvvdgftlrwvlsdtlkqkflllckqcksvlccrvspaqkaavvsmvkngldvmtlsigdgandvamiqeadvgvgiageegrqavmssdyaigqfrfltrlvlvhgrwsyrrlaetisnffyknmvwtwaifwfqifcdfdisyifdytyilmfnlfftsipvilmgvldqdvsdtvslavpqlyrrgierlewtqtkfwaymadgiyqsvmsffipfifciltpaasgngldvqertrlgcyiahpavltinmyilmntyrwdwvmllvvflsdififfwtgiytatsysgqfyqaapqvyaeftfwmafiitpticllprlvtkciqkqmfpydvdiireristgeykaievpvvapvedealkgtssgssessssraksksskhskhthyasvdedrrpiyppsvnthhtraqngsdgtnytrhsldtnqiedeppmgggrpsidrarpsydrlrrsmdrerirqsleqsndftsaarlsriesthsaqnlptpgaqrrfnlttvrkrglsvfskqsedevphpgvededkrderrdsqq

>NcDnfB

maagrpppgapssaqhddllldfgneqpyygggqrstlndddllrlhsddaqgaaqprpsvsyddfvgsgqttqptatrpiqpapgatggggpispypdsrsldrhfsqtsdlgnyqryaedsddypdesttsyyqhggaippdsaaardsararnsvlsmgggligraknmlgmgpegysemdlpltdpratarvdhsheppppqkpakkfdfkfgfgggkpdpatlgpriihlnnppanslnkyvdnhvstakynfatflpkflfeqfskfanifflftaglqqipglsptnryttigplavvllvsagkemvedyrrkqadkalnmskarilrgstfeetkwinvsvgdiirveseesfpadlvllassepeglcyietanldgetnlkikqalpetssmvssselsrlggrirseqpnsslytyeatltmqagggekelplnpeqlllrgatlrntpwvhgvvvftghetklmrnataapikrtkverqlntlvlflvgillifsvvstvgdliqrkvegeeglaylfldpmngasavarifikdmvtywvlfsalvpislfvtiemvkywhgilinddldmyydvndtpancrtsslveelgmvefvfsdktgtltcnmmeyrqcsiagimyadkvpedripsgedgedgihdfkqlqknleshqsaqvidqfltllaichtvipeqaedgsikyqaaspdegalvdgavqmgyrfvarkpraviieangqqleyellavcefnstrkrmstiyrcpdgkvrcyckgadtvilerlndqnphvdatlrhleeyaseglrtlclamreipehefqewmkvyetaqttiggnradeldkaaeliehdfyllgataiedrlqdgvpetihtlqeagikvwvltgdrqetainigmsckllsedmmllivneesaeatrdnlqkkldairnqgdatiemetlalvidgksltyalekdmeklfldlaimckaviccrvsplqkalvvklvkkyqkesillaigdgandvsmiqaahigvgisgmeglqaarsadvsiaqfrylrklllvhgawsyhrvsktilfsfykniclyltqfwytfqnvfsgeviyeswtlsfynvfftvlpplalgildqfvsarlldrypqlynlgqrntffkirvfgewiinavyhsiilyvggclfwlndgpqgdgfpggkwvwgtamygavlltvlgkaalvtnnwtkyhviaipgsmafwilfvavygevapklnisveyfgviprlftspifwiqmptlailcllrdfawkfskrlwrpeayhhvqeiqkyniqdyrprmeqfqkairkvrqvqrmrkqrgyafsqadesqtrvlqaydttqhrgrygemassrpnq

>NcDnfC1

magpdqpdaaealrdrlenielqqnevhiprpalrrlpastpslrlgqrrstdgrhpavhgrpslglprhsshlapetptsplspaspsatrvrfsldipdddlprsgrpslsrpslqlpsqlytinsnidlpsssvkpsqpgrpsleprhsaanstrsrlsltlprqrydeaygdsssamateeetkitldtykdwigrqkerqkrltsdplnrikagaenayqkyivegllrqrpippskdgrhiplkpglvrqtplndertgqpyvsnfirssrytlydflpkqllfqfgklanfyflvigilqmvpglsttgtyttiaplivfvcismakegyddyrryrldklenrsaafvldpdgtvstrvrgrkrgmlmemvkgkksaeggaatelaglervttaevdqsegegggepwariewqdirvgdivqlrrdenvpadmvllhatgpngvayietmaldgetnlkskqacplllnhcsstndmahcdatvvsedpnldlynyegrvtvngetmpltsnqivyrgstlrnttqaigivvntgeeckirmnahknvrtkapemqfmvnrivmllvvfvvalavgctggytmwhrsnerkawyliqqkvpikeivigfiiafntliplslyvsleiiklgqlylladvemydpktdtamsantttilenlgqvsyvfsdktgtltenlmrfrkisvggaaflhdmdirrdaekakqeqlaqdlpkrslsisakprpsnveasleplaeessnepivanqsawhssagvkhgqrepnteelirylqqrpntpfsriakhfilcialchtclpetkedgemtfqaaspdelalvegardlgylvidrpsqairlqtrdadgnpvtetyevldviefsskrkrmsivirmpdgstciftkgadsmilprlkqsnlaiqtagkigqmasmrksmeqekalrrlslhsikrdsmnlvrasrasmdhrrvkgsqrrstfftdevdswlvrretdefddsnppprrsmqrgrsfevgrphdpldgmvdehlalneaavfercfkhiddfaseglrtlvfayryldgeeyrkwktiyheattslvnrqerieaaaeiieqnfdlagataiedklqhgvpetidkfrranikiwmltgdkretainiahsarickpfsevyildatqgdlqerlastlidvgrgmvphsvvvidghtlsvveedeslrvlffdlvvrvdsviccraspsqkatlvqsirqqvpkavtlaigdgandiamiqashvgigisgreglqaarisdfsisqfrflqrllfvhgrwnyirtgkyilgtfwkeivfylvqaqfqryngytgtslfestsltvfntlftslpvilfgifekdleadtlmaipelytygqkekafnvrlyigwmfmavsesvlifsivwyvygitlppenaalypvgtlafticvifinikmlvlelhnriaicfaglfisvfgwflwnlllsgifsakmspylvrdgfirgfgqhapwwvvciialsallifevaisavrrslwptdqdlmkeiehidgvlevmkehaaergeaglpaadvtaaeagsmrhknsygdftgasmtpaaagssssrplmpegrpshaytrpnfdspaeeledpvedvvrlkskaagevgepkgsmkgktprksalrtnmdvgvstrflgegsantpiasgsameasepyfvtspiempemhspvvstaaaatadtgvgseeagegsaverqltgttvnqeegqppvtrqll

>NcDnfC2

mrsftrstadegggasvdnasfekglsifsktgilaplrktgtsattltrtikastqqlyrryvlelifqekelppskhgrhiplrphhdkpliderrgsayipntirtsrytiydfipkqlffqfsrlanfyflcvgvpqtipgvsttgnfttiipllffvfltivkegyddwkrhrldkvenarsatvlrptghvdsrqdwrtgfglrrtkdkdtvavqevipdseyqwhatqwkdlkvgdviklsrdedvpadivllyadgensmayietmaldgetnlknkqvskalqrcntiqgiancqaefvvedpnpdlyrfdgrvtvdgetlpltlnevvyrgctlrnttcaigmvintgeecklrrnadrhpkakkpamekianrivislvvvvislsigcsmgyllwknayehkawylrhlgvdfediiigffiqfnniiplalyvsleivrigqmwwlnsdlemydkvsdtpakcntntilenlgqigyvfsdktgtltenvmkfrkmsiagttwlhemdivkeqeaqgltqtetkgkglnvmleptfendnaltktttavsvrspnprrsssqwrstgrpdhvqpevttedlleyirlrpkapfsrravqyllamalchtclpeirdgeiefqaaspdelalvkaaqelgflvvqrssqsvtlrisrgdgtelertyqildviefsskrkrmsiivrcpderiwlitkgadsvilprlrmaqlaiqkanevrkslevehemqrrseareprnsfggrpsltirrsihlarqnsvagpskrpitnrsksfevgrmslsapenrlrpqvtvrtasydvpyedaaaasvsdkfaflddpsvcdegaiftrcfkhiddfateglrtllfaqrflseseyntwkklyrdaetslvdrqerietagelieqtldligataiedklqkgvpetierlrranikiwmltgdkretainiahsarlcrptsdiyildatkgnlegqimdivdelniraetmptaipnhtvvvidghtlaaldqpethgnakelfysliptidsviccraspaqkallvtairnhshqpsttkkkgflaylitprrtppltlaigdgandlamlsaahvgvgisgreglqaarvadyavsqfrflarlllvhgrwnyartskfivatfwkemffylptelyqrytgytgtslyeswsltvlntlftslcvivpgvweqdlsaetlmavpelyvwgqrdgglnlrkylgwmvaavaqgvvvwwvcwglyggiavkdnglfavgnlvftvaivwtnlklliidthhktgiilgafgitfagwwiwqiflasayapgvwpyavrggffssfgpdpawwvalfaavglltavelgykstkrnmiisglwrlgwrkwmqwetwkgllalgrgggargagpmwawdeaggegrnveewdvelwqalereqtvrealkgmarvghecdgpepeeiskeeedneknrveesstmdsegqhesavveslqisk

>NcDnfD

mpssdtyhlskppdshvvdesdsdleidleeldpqatdpnnhhqslgqhrsrnsseqrppppttsialrnlrmgslrrgakrngygelgrsrdgnddddadallrhdrhsvgsathdedagllsehggggrirtssgrrrrsfagdglrnismklpgfmsgdqsnhndeqeqeeddpsssrlvavgssqstrfppniisnakytawsflpvtlynefsfffnmyfllvalsqaipalrigylstyvaplafvlfitlakeayddierrrrdneanseeytvlqfddpgaslginrprrkmksshtrtgskrlgieqendrlsdiqeeeeqtegrglrelpasylsevskksrdlkvgdvlkltkghrvpadviilkclahesaanketeeqevpakeemllldhvddddvgegssknaktskesnnngaegsssgetfirtdqldgetdwklrlasplsqnlsteelvrlrvtagkpdkrvnefvgtlellpsrqdvmsgaaynpregddvkaaplsidntawantviasnattlavivytgpqtrsalstspsrsktglleyeinsltkilcfltlflsivlvalegfstakgniwyvkimrflvlfstivpislrvnldlgksvyswfiqrdpgipgavvrtstipedlgrieyllsdktgtltqnememkkihvgtvsyaneamdevatyvkqgfnlsssassaslalatpsstysaanvgatrtrreigsrvrdvvlalalchnvtptseedenghtvnsyqasspdeiaivkwtesvglrlahrdrknivlesaetgrpvvkvrildifpftsegkrmgiivqfyeklqpgnpslgsseiwfyqkgadtvmssivaandwldeetanmareglrtlvvgrkrlslkeyqefsakhheaslaisgreanmqavvsqylehnlellgvtgvedklqkdvkpslellrnagikiwmltgdkvetarcvavssklvargqyihtiaklrrkdgaqdnldllrsktdscllidgeslamylthfrrefisvavllptvvacrcsptqkaevaklikeytkkrvccigdggndvsmiqaadvgvgivgkegrqaslaadfsieqfhhltkllvwhgrnsykrsaklaqfvihrgliiavcqtmysialkfepeglykdwlmvgyatvytafpvlslvldkdvdedlanlypelykeltsgsslsyrtffvwvfvsiyqgcliqglsqvltgidgprmlavsytvlvlnellmvaieittwhwvmvvsivgtflmyivsipflgdyfdlkfvltlgflwrvaaiaaisliptyaakvirrtmkppsyrkvqgi

>ScDnf1

MSGTFHGDGHAPMSPFEDTFQFEDNSSNEDTHIAPTHFDDGATSNKYSRPQVSFNDETPKNKREDAEEFTFNDDTEYDNHSFQPTPKLNNGSGTFDDVELDNDSGEPHTNYDGMKRFRMGTKRNKKGNPIMGRSKTLKWARKNIPNPFEDFTKDDIDPGAINRAQELRTVYYNMPLPKDMIDEEGNPIMQYPRNKIRTTKYTPLTFLPKNILFQFHNFANVYFLVLIILGAFQIFGVTNPGLSAVPLVVIVIITAIKDAIEDSRRTVLDLEVNNTKTHILEGVENENVSTDNISLWRRFKKANSRLLFKFIQYCKEHLTEEGKKKRMQRKRHELRVQKTVGTSGPRSSLDSIDSYRVSADYGRPSLDYDNLEQGAGEANIVDRSLPPRTDCKFAKNYWKGVKVGDIVRIHNNDEIPADIILLSTSDTDGACYVETKNLDGETNLKVRQSLKCTNTIRTSKDIARTKFWIESEGPHSNLYTYQGNMKWRNLADGEIRNEPITINNVLLRGCTLRNTKWAMGVVMFTGGDTKIMLNSGITPTKKSRISRELNFSVVINFVLLFILCFVSGIANGVYYDKKGRSRFSYEFGTIAGSAATNGFVSFWVAVILYQSLVPISLYISVEIIKTAQAAFIYGDVLLYNAKLDYPCTPKSWNISDDLGQVEYIFSDKTGTLTQNVMEFKKCTINGVSYGRAYTEALAGLRKRQGIDVETEGRREKAEIAKDRDTMIDELRALSGNSQFYPEEVTFVSKEFVRDLKGASGEVQQRCCEHFMLALALCHSVLVEANPDNPKKLDLKAQSPDEAALVATARDVGFSFVGKTKKGLIIEMQGIQKEFEILNILEFNSSRKRMSCIVKIPGLNPGDEPRALLICKGADSIIYSRLSRQSGSNSEAILEKTALHLEQYATEGLRTLCIAQRELSWSEYEKWNEKYDIAAASLANREDELEVVADSIERELILLGGTAIEDRLQDGVPDCIELLAEAGIKLWVLTGDKVETAINIGFSCNLLNNEMELLVIKTTGDDVKEFGSEPSEIVDALLSKYLKEYFNLTGSEEEIFEAKKDHEFPKGNYAIVIDGDALKLALYGEDIRRKFLLLCKNCRAVLCCRVSPSQKAAVVKLVKDSLDVMTLAIGDGSNDVAMIQSADVGIGIAGEEGRQAVMCSDYAIGQFRYLARLVLVHGRWSYKRLAEMIPEFFYKNMIFALALFWYGIYNDFDGSYLYEYTYMMFYNLAFTSLPVIFLGILDQDVNDTISLVVPQLYRVGILRKEWNQRKFLWYMLDGLYQSIICFFFPYLVYHKNMIVTSNGLGLDHRYFVGVYVTTIAVISCNTYVLLHQYRWDWFSGLFIALSCLVVFAWTGIWSSAIASREFFKAAARIYGAPSFWAVFFVAVLFCLLPRFTYDSFQKFFYPTDVEIVREMWQHGHFDHYPPGYDPTDPNRPKVTKAGQHGEKIIEGIALSDNLGGSNYSRDSVVTEEIPMTFMHGEDGSPSGYQKQETWMTSPKETQDLLQSPQFQQAQTFGRGPSTNVRSSLDRTREQMIATNQLDNRYSVERARTSLDLPGVTNAASLIGTQQNN

>ScDnf2

MSSPSKPTSPFVDDIEHESGSASNGLSSMSPFDDSFQFEKPSSAHGNIEVAKTGGSVLKRQSKPMKDISTPDLSKVTFDGIDDYSNDNDINDDDELNGKKTEIHEHENEVDDDLHSFQATPMPNTGGFEDVELDNNEGSNNDSQADHKLKRVRFGTRRNKSGRIDINRSKTLKWAKKNFHNAIDEFSTKEDSLENSALQNRSDELRTVYYNLPLPEDMLDEDGLPLAVYPRNKIRTTKYTPLTFFPKNILFQFHNFANIYFLILLILGAFQIFGVTNPGFASVPLIVIVIITAIKDGIEDSRRTVLDLEVNNTRTHILSGVKNENVAVDNVSLWRRFKKANTRALIKIFEYFSENLTAAGREKKLQKKREELRRKRNSRSFGPRGSLDSIGSYRMSADFGRPSLDYENLNQTMSQANRYNDGENLVDRTLQPNPECRFAKDYWKNVKVGDIVRVHNNDEIPADMILLSTSDVDGACYVETKNLDGETNLKVRQSLKCSKIIKSSRDITRTKFWVESEGPHANLYSYQGNFKWQDTQNGNIRNEPVNINNLLLRGCTLRNTKWAMGMVIFTGDDTKIMINAGVTPTKKSRISRELNFSVILNFVLLFILCFTAGIVNGVYYKQKPRSRDYFEFGTIGGSASTNGFVSFWVAVILYQSLVPISLYISVEIIKTAQAIFIYTDVLLYNAKLDYPCTPKSWNISDDLGQIEYIFSDKTGTLTQNVMEFKKCTINGVSYGRAYTEALAGLRKRQGVDVESEGRREKEEIAKDRETMIDELRSMSDNTQFCPEDLTFVSKEIVEDLKGSSGDHQQKCCEHFLLALALCHSVLVEPNKDDPKKLDIKAQSPDESALVSTARQLGYSFVGSSKSGLIVEIQGVQKEFQVLNVLEFNSSRKRMSCIIKIPGSTPKDEPKALLICKGADSVIYSRLDRTQNDATLLEKTALHLEEYATEGLRTLCLAQRELTWSEYERWVKTYDVAAASVTNREEELDKVTDVIERELILLGGTAIEDRLQDGVPDSIALLAEAGIKLWVLTGDKVETAINIGFSCNVLNNDMELLVVKASGEDVEEFGSDPIQVVNNLVTKYLREKFGMSGSEEELKEAKREHGLPQGNFAVIIDGDALKVALNGEEMRRKFLLLCKNCKAVLCCRVSPAQKAAVVKLVKKTLDVMTLAIGDGSNDVAMIQSADVGVGIAGEEGRQAVMCSDYAIGQFRYVTRLVLVHGKWCYKRLAEMIPQFFYKNVIFTLSLFWYGIYNNFDGSYLFEYTYLTFYNLAFTSVPVILLAVLDQDVSDTVSMLVPQLYRVGILRKEWNQTKFLWYMLDGVYQSVICFFFPYLAYHKNMVVTENGLGLDHRYFVGVFVTAIAVTSCNFYVFMEQYRWDWFCGLFICLSLAVFYGWTGIWTSSSSSNEFYKGAARVFAQPAYWAVLFVGVLFCLLPRFTIDCIRKIFYPKDIEIVREMWLRGDFDLYPQGYDPTDPSRPRINEIRPLTDFKEPISLDTHFDGVSHSQETIVTEEIPMSILNGEQGSRKGYRVSTTLERRDQLSPVTTTNNLPRRSMASARGNKLRTSLDRTREEMLANHQLDTRYSVERARASLDLPGINHAETLLSQRSRDR

>ScDnf3

mgiadgqrrrssslrtqmfnkhlydkyrgrtddeieledinesktfsgsdnndkddrdetsgnyaaeedyemeeygspdvsysiitkildtildrrrtfhskdgrhipiildhnaieykqaatkrdghliderfnkpycdnritssrytfysflprqlyaqfsklantyffivavlqmipgwsttgtyttiiplcvfmgismtreawddfrrhrldkeennkpvgvlvkdgnndaqevytlpssvvsstayltksaaaennpplnddrnssqghfldthfnnfellknkynvhihqkkweklrvgdfvlltqddwvpadlllltcdgensecfvetmaldgetnlkskqphpelnkltkaasglaninaqvtvedpnidlynfegnlelknhrndtimkyplgpdnviyrgsilrntqnvvgmvifsgeetkirmnalknprtkapklqrkinmiivfmvfvvatislfsylghvlhkkkyidqnkawylfqadagvaptimsfiimyntviplslyvtmeiikvvqskmmewdidmyhaetntpcesrtatileelgqvsyifsdktgtltdnkmifrkfslcgsswlhnvdlgnsednfednrdntnslrlppkahngssidvvsigdqnvldrlgfsdapiekghrpsldnfpksrnsieykgnssaiytgrpsmrslfgkdnshlskqasvispsetfsenikssfdliqfiqryptalfsqkakffflslalchsclpkkthnesigedsieyqssspdelalvtaardlgyivlnrnaqiltiktfpdgfdgeaklenyeilnyidfnsqrkrmsvlvrmpnqpnqvllickgadnvimerlhdrelaakkmadictstkerkdaeaelvlqqrkslermvdeeamartslrnslssvpraslslqavrkslsmknsrtrdpekqidsidqfletvkksdqeigsvvnksrkslhkqqiekygprisidgthfpnnnvpidtrkeglqhdydteilehigsdelilneeyviertlqaidefsteglrtlvyaykwidigqyenwnkryhqaktsltdrkikvdeagaeiedglnllgvtaiedklqdgvseaiekirragikmwmltgdkretainigyscmlikdystvviltttdeniiskmnavsqevdsgniahcvvvidgatmamfegnptymsvfvelctktdsvic

craspsqkalmvsnirntdpnlvtlaigdgandiamiqsadigvgiagkeglqasrvsdysigqfrfllkllfvhgrynyirtskfmlctfykeitfyftqliyqrytmfsgsslyepwslsmfntlftslpvlcigmfekdlkpmtlltvpelysygrlsqgfnwlifmewvilattnsliitflnvvmwgmsslsdntmyplglinftaivalinvksqfvemhnrnwlaftsvvlscggwlvwccalpilnntdqiydvaygfynhfgkditfwctslvlallpitldivyktfkvmiwpsdsdifaeleqksdirkklelgaysemrqgwtwdkdpstftrytdkvlsrprtnsrasakthnssiysmsngnvdhsskknffgnsskksseryevlpsgklikrpslktqsskdsiggnittkltkklklpsrnvededvnqiiqarlkdle

>ScDrs2

MNDDRETPPKRKPGEDDTLFDIDFLDDTTSHSGSRSKVTNSHANANYIPPSHVLPEETIDLDADDDNIENDVHENLFMSNNHDDQTSWNANRFDSDAYQPQSLRAVKPPGLFARFGNGLKNAFTFKRKKGPESFEMNHYNAVTNNELDDNYLDSRNKFNIKILFNRYILRKNVGDAEGNGEPRVIHINDSLANSSFGYSDNHISTTKYNFATFLPKFLFQEFSKYANLFFLCTSAIQQVPHVSPTNRYTTIGTLLVVLIVSAMKECIEDIKRANSDKELNNSTAEIFSEAHDDFVEKRWIDIRVGDIIRVKSEEPIPADTIILSSSEPEGLCYIETANLDGETNLKIKQSRVETAKFIDVKTLKNMNGKVVSEQPNSSLYTYEGTMTLNDRQIPLSPDQMILRGATLRNTAWIFGLVIFTGHETKLLRNATATPIKRTAVEKIINRQIIALFTVLIVLILISSIGNVIMSTADAKHLSYLYLEGTNKAGLFFKDFLTFWILFSNLVPISLFVTVELIKYYQAFMIGSDLDLYYEKTDTPTVVRTSSLVEELGQIEYIFSDKTGTLTRNIMEFKSCSIAGHCYIDKIPEDKTATVEDGIEVGYRKFDDLKKKLNDPSDEDSPIINDFLTLLATCHTVIPEFQSDGSIKYQAASPDEGALVQGGADLGYKFIIRKPNSVTVLLEETGEEKEYQLLNICEFNSTRKRMSAIFRFPDGSIKLFCKGADTVILERLDDEANQYVEATMRHLEDYASEGLRTLCLAMRDISEGEYEEWNSIYNEAATTLDNRAEKLDEAANLIEKNLILIGATAIEDKLQDGVPETIHTLQEAGIKIWVLTGDRQETAINIGMSCRLLSEDMNLLIINEETRDDTERNLLEKINALNEHQLSTHDMNTLALVIDGKSLGFALEPELEDYLLTVAKLCKAVICCRVSPLQKALVVKMVKRKSSSLLLAIGDGANDVSMIQAAHVGVGISGMEGMQAARSADIAVGQFKFLKKLLLVHGSWSYQRISVAILYSFYKNTALYMTQFWYVFANAFSGQSIMESWTMSFYNLFFTVWPPFVIGVFDQFVSSRLLERYPQLYKLGQKGQFFSVYIFWGWIINGFFHSAIVFIGTILIYRYGFALNMHGELADHWSWGVTVYTTSVIIVLGKAALVTNQWTKFTLIAIPGSLLFWLIFFPIYASIFPHANISREYYGVVKHTYGSGVFWLTLIVLPIFALVRDFLWKYYKRMYEPETYHVIQEMQKYNISDSRPHVQQFQNAIRKVRQVQRMKKQRGFAFSQAEEGGQEKIVRMYDTTQKRGKYGELQDASANPFNDNNGLGSNDFESAEPFIENPFADGNQNSNRFSSSRDDISFDI

>ScNeo1

MPNPPSFKSHKQNLFNSNNNQHANSVDSFDLHLDDSFDAALDSLQINNNPEPLSKHNTVGDRESFEMRTVDDLDNFSNHSSDSHRKSSNTDTHPLMYDNRLSQDDNFKFTNIASSPPSSSNNIFSKALSYLKVSNTKNWSKFGSPIELSDQHIEREIHPDTTPVYDRNRYVSNELSNAKYNAVTFVPTLLYEQFKFFYNLYFLVVALSQAVPALRIGYLSSYIVPLAFVLTVTMAKEAIDDIQRRRRDRESNNELYHVITRNRSIPSKDLKVGDLIKVHKGDRIPADLVLLQSSEPSGESFIKTDQLDGETDWKLRVACPLTQNLSENDLINRISITASAPEKSIHKFLGKVTYKDSTSNPLSVDNTLWANTVLASSGFCIACVVYTGRDTRQAMNTTTAKVKTGLLELEINSISKILCACVFALSILLVAFAGFHNDDWYIDILRYLILFSTIIPVSLRVNLDLAKSVYAHQIEHDKTIPETIVRTSTIPEDLGRIEYLLSDKTGTLTQNDMQLKKIHLGTVSYTSETLDIVSDYVQSLVSSKNDSLNNSKVALSTTRKDMSFRVRDMILTLAICHNVTPTFEDDELTYQAASPDEIAIVKFTESVGLSLFKRDRHSISLLHEHSGKTLNYEILQVFPFNSDSKRMGIIVRDEQLDEYWFMQKGADTVMSKIVESNDWLEEETGNMAREGLRTLVIGRKKLNKKIYEQFQKEYNDASLSMLNRDQQMSQVITKYLEHDLELLGLTGVEDKLQKDVKSSIELLRNAGIKIWMLTGDKVETARCVSISAKLISRGQYVHTITKVTRPEGAFNQLEYLKINRNACLLIDGESLGMFLKHYEQEFFDVVVHLPTVIACRCTPQQKADVALVIRKMTGKRVCCIGDGGNDVSMIQCADVGVGIVGKEGKQASLAADFSITQFCHLTELLLWHGRNSYKRSAKLAQFVMHRGLIIAICQAVYSICSLFEPIALYQGWLMVGYATCYTMAPVFSLTLDHDIEESLTKIYPELYKELTEGKSLSYKTFFVWVLLSLFQGSVIQLFSQAFTSLLDTDFTRMVAISFTALVVNELIMVALEIYTWNKTMLVTEIATLLFYIVSVPFLGDYFDLGYMTTVNYYAGLLVILLISIFPVWTAKAIYRRLHPPSYAKVQEFATP

>VdDnf1

MASASTNLNPGFLAPPPTEGVGAGAGQTSSSGDAPESSDPNQPGPADAKTQRSRWPTRKMTLKSSNNKRLSILGRANKKHNGNEKNRLSGDPNNVNAESQGSEAEEHGAGRKLYFNLDLPREMVDEDGAPIQQYARNKIRTAKYTPMSFVPKNLWFQFHNIANIFFLFVVVLVVSCTHSPVTVPTLVDHNHQQIRAL.FFPIFGGYNPGLNSVPLIAIITITAIKDAIEDYRRSNLDNELNNAPVHRLRGFNNINVQEDNVSAWRKFKKANSALFGSLWHAMQTIWSKKAREDREKQRLARQEAEMPRRSIETQRQSFMSARDEDIQMTPVPSPMPRQNLALPDEHDERRAHQQKQLKGDVINRELPVKGSARFHRDAWKDLRVGDYVRIYNDDEIPADIVILATSDPEGACYVETKNLDGETNLKFRSALRCTRSMKHARDAERAQFWMDSEAPQANLYKYNGAINWQQKFDGFDSEPHNMVEPITIDNMLLRGCNLRNTDWALGIVMFTGHDTKIMINSGITPSKRARIARELNYNVIWNFGILVVMCLTAAIVNGTSWARTDRSLSFFNYGSIGGSAPMTGFITFWAAMIFFQNLVPISLYITLEIVRLLQAIFIYSDVEMYYAPIDQPCIPKSWNISDDLGQIEYIFSDKTGTLTQNVMEFKKATINGQPYGEAYTEAQAGMQKRMGIDVEKEGERARAEIAEGKVRSLEGLRRIHDNPYLHDEDLTFIAPDFVADLAGESGPEQQAANEHFMLCLALCHTVIAERPPSDPPKLVFKAQSPDEAALVATARDMGFTVLGTSAEGVNLNVMGEERHYPIMNTIEFNSSRKRMSIILRMPDGRILLICKGADSVIYSRLRRGEQAELRRSTGEHLEMFAREGLRTLCIAQRELSEDQYSAWLEEHNAAAAALDDREEKLEAVADRLEQDLTLLGGTAIEDRLQDGVPDTIALLGQAGIKLWVPTGDKVETAINIGFSCNLLNNDMELIHLKIEEDETGDTPDDVFLTRVDELLDTHLQTFGMTGSDEELVKARDNHEPPDATHGLVIDGFTLKWVLHESLKQKFLLLCKQCKSVLCCRVSPAQKAAVVSLVKNGFDVMTLSIGDGANDVAMIQEADVGVGIAGVEGRQAVMSSDYAIAQFRFLQRLVLVHGRWSYRRLAESISNFFCKNVIWTFALFWYQIYCDFDITYIFDYTYILFFNLFYTSVPVAIMGVLDQDVSDKVSLAVPELYRRGIERREWTQTKFWYVLQVFMTLGACKTNSDAGSTWWTASTNRSWLSGFLTLWYQQASSPLMVRISKTARDWAPTSPTPLSSQSTCTF.STHTGGIGSSCCAPSSATP.SSRRLASSRRRPLQVPFTAPARRSTARPRSGPSYSSCPSSASFPVSRSRHCRRCTSPTTSTLSASKNDRASSRTSTRHRWIRKSMPQRPDDRAPERRRRLIASQRVSTCTMPASTKTEDQSIPPQLRRVRRTTHEARTVAMARTTRGTACRWTWRLRFGLLSTELAHPTTESAHRWTASGPASRQAAISRRLHGCHESSRASLPVTSSTDASAGCPRPRAPIN

>VdDrs2

MAGRPTGGPQGAPSHDLLLDLENDQPVYGGGQRSTLNDDDLMRTYTRDQESGQDQGRPSVSYDDFIGAGQSRQHGTGSQPGGPGQSSSSNNNNNNVSAPYSRSGRQYSQTSDLGNYQRYADDFDDYPADGDSFYQQGGALNGGGADAAARHNARNRNSVLTMGGGFFGKMKNRLGMGQGYSEMDLPLTEPGGGGGGAGGGGHSRADSSGIDPPKRDKKFDMGNFKFGFGRSKPDPSTLGPRIIHLNNPPANAANKYVNNHVSTAKYNIATFLPKFLLEQFSKIANVFFLFTAALQQIPGLSPTNRFTTIIPLVAVLMVSAGKELVEDYRRKQADAALNTSRAQVLRGSTFEETKWINVAVGDIVRVESEEPFPADIVLLASSEPEGLCYIETANLDGETNLKIKQALPETSQMVSSSELSRLGGWMKSEQPNSSLYTYEATLTMQTGGGEKELPLNPEQLLLRGATLRNTPWIHGVVVFTGHETKLMRNATAAPIKRTKVEKKLNTLVLLLVGILMVLSIISTVGDLIIRRVEGDAISYLMLDQPDTAGKIAETFFKDMVTYWVLFSSLVPISLFVTVEMVKYWHGILINDDLDMYYDRNDTPANCRTSNLVEELGMVEFVFSDKTGTLTCNMMEFKQASIAGIQYADEVPEDRRATIQDGVEVGLHDYKRLKENRKNHSSAPAIDHFLALLATCHTVIPEKGDEKGGKIKYQAASPDEGALVDGAATLGYTFTDRKPKAVFIEVDGQTLEYELLAVCEFNSTRKRMSTIYRCPDGVIRVYCKGADTVILERLNENNPHVEQTLTHLEEYASEGLRTLCLAMREVSEQEFQEWNQVYEKAATTVGGNRAEELDKASEMIEHDFFLLGATAIEDRLQDGVPETIHTLQEANIKVWVLTGDRQETAINIGMSCKLLSEEMMLLIINEESAAATRDNIEKKLEAIRAQGDRTIELETLALVIDGKSLTYALEKDLEKMFLDLAIMCKAVICCRVSPLQKALVVKLVKKYQKESILLAIGDGANDVSMIQAAHIGVGISGEEGLQAARSADVSIAQFRFLKKLLLVHGAWSYQRVAKTILYSFYKNITLYMTQFWVCPPAPMHKDPPLTSFSTHSATSSQVRSFTNHGHSPFITSSIPFFLPWPSAFLISPSRRASSIGTLNCTAWASRTSFSV.RYSSNGSSTPSTTPSSFTSLASSSGTAT.YSRTVRSPATGCGVPPSTHPSSSRSSARPALSPATGPSTMSLPFPAPWPFGGSSSPSTAPSPP.SPFLPSSTASFPSCTAAPSSGFKALLLPSCACCGTLRGSMPSACTGQNRIITSRRFKSTISKIIDLGMSSPLTAQHEHDLIRDQHGTIPKGHSQGSPGAAYAEAAWLRFLAGGRESGARASGLRYDAEPWPIRRDDELAATRTGNL

>VdNeo1

MPPNSSYRPAEPPDSPNDSDSDLDLDIQELDPATTSQRGPAQARHARDTGESRSPRIALRNLRMGGLRRGNRSRGYGELGQDRDGAADDSQSLLGGGHRRSTSQSADGSRASGDDAPLLSGRSQPRSRRRSSAQEGLGRLAALGSSLKLASFMGGSAGPGSVKDDSGEAEEEDDPSAARHIAVGSLQSTKFPPNLISNAKYTALSFLPVTLYNEFSFFFNMYFLLVALSQAIPALRIGYLTTYVAPLAFVLMITMGKEAYDDIERRRRDNEANAEAYTVLRFDEPGRALASELRPHKRLKSESTKRRGKKLVKTADRLSDIREEEERMEGDGPRTGPTSSVYEMPCKSRDLKVGDVLKLSKGQRVPADVVILKCITAESAQVEEEEAPVEEESLLVDALDDAAPAASSKGKDVENKAEDASGSNGETFIRTDQLDGETDWKLRLSSPLTQNLPTEELVRLRVTGGKPDRKVNEFMGALQLLPTREDAQSTSADHGFGADAGTTAPLSIDNTAWANTVIASQATTLAVIMYTGTQTRSALSTAPSRSKTGLLENEINSLTKILCALTLALSVILVALLGFENTEDNRWYIKIMRYLVLFSTIVPISLRVNLDMGKSAYSWFIHRDSGIPGTVVRTSTIPEDLGRIEYLLSDKTGTLTQNEMEMKKIHVGTVSYANEAMDEVVAYVKQGFHIQSTVDPASQKMLITPSSTFASTANVGTTRTRREIGSRVRDVVLALALCHNVTPTTEEEDGKTITSYQASSPDEIAIVRWTESVGLRLVHRDRTGMILEYAETGRPTVQVRILDVFPFTSEGKRMGIIVQFYETKQSSPPDLSSGEIWFYQKGADTVMTSIVAENDWLDEETANMAREGLRTLVVGRRRLSYEQYREFSRSHQEAALAITGRDANMQKVVSQYLERDLELLGVTGVEDKLQKDVKPSLELLRNAGVKIWMLTGDKVETARCVAVSSRLVARGQYIYTVSKLKRKDNAQDHLDFLRSKTDACLLVDGESLLLFLTHFRIEFISVAVQLPTVVACRCSPTQKAEIATLIKEYTKKRVCCIGDGGNDVSMIQAADVGVGIVGKEGRQASLAADFSIEQFHYLTKLLVWHGRNSYKRSAKLAQFVIHRGLIIAVCQTMYSIAIKFEPQGLYKDWLLVGYATVYTAFPVLSLVLDKDVDENLANLYPELYKELTSGRSLSYRTFLIWVLVSVYQGCVIQGLSQILTEVHGPRMVAVSYTVLVLNELLMVAMEITTWHPVMIVSIVGTFMMFAASIPFLGDYFELEFLLTLGFYWRVLAIGAISLIPPYAAKLIGRALKPPSYRKVQST

>VdP4-4

MARPGPRVPAFSSYSTIDDDGPNPNDPSNLTGTTSDTRSRRPRHAERANTEPMRSAQSIDGAATPQIRVSSDGLQEDLARQQPYISDSPAHRRSQVTMTGGDPTKNVDVAVSHSTIDDEPKTPLPSQRPHLTKRMQWLDRRDAAAARARMLYQKVVIEGILRQKPLPPSADGRHIPITPGRKRADLLTDERAQKPYTGNFIRSSRYTVWDFLPKQLIFQFSKLGNAYFLVISIMQMIPGLSTTGRWTTIAPLMAFVSLSMAKEGYDDYRRYLLDKADNRSEVLVLGEDMRALHRVKNKTKKHGGGKDGGDELPMHDMGESQVARDGDWSLIQWQDVKVGDIVRLRRDENVPADIVLLHATNPHSVAYIETMALDGETNLKAKRALPMLAEQCGTAQGLKACDATVVSEDPNIDLYRYDGRVTMKEETMPLSLNNVVYRGSVVRNTTETIGLVVNTGEECKIRMNASKHVHAKAPRMQSVANRCIIWLVFVLLALSGGLTGGYYIWRDPQEDDSWFLGGRTLSFKEVFIAYIIMFNTLIPLSLYISLEIIKVIQFYLMGDVEMYDPVTNTPMVANTTTILEDLGQVNYVFSDKTGTLTENVMRFRKMSVAGTAWLHDMDVERDEEAKQKLIDMARKKRKGKDKQKFEKSKAIETNPASTQPGRQSMSSTRWKASTARAEDEPELKTEELLGYLRRKPNTPYSKKAKQFLLCLALCHTCIPEVGKDGQTEYQSASPDEQALVEAARDLGYVMIDRPNQEVILQLLGPDGNIVRETYQVLDVIDFTSKRKRMSIIIRMPDGRICVFCKGADSIILPRLKLAHLAMQKASAVELKASKHRSMEQGKEQRRISNSTPRTSMALGRPSMSKRHSHLLDGHRMSIEGSRRGSALVEGSSPWGTRRGSLEDQDFHTPLQSPIGLMHSPRPSTTFSRNDDALDGLVDESAALNEGTIFERSFQHIDDFARDGLRTLLYGFRYLDEGEYTKWRAIYAEATTSLDNRQEKIEAAGELIEEKFELAGVTAIEDKLQEGVPETIDKLRRAEIKVWMLTGDKRETAINIAHSARLCKPFSELFILDATTGPINESISAALAEVGRGMLPHTVVVVDGQTLSTIDNDEGLSYLFYDLAGRVDSVICCRASPAQKANLVKRIRRQVPKSLTLAIGDGANDIGMIQASHVGIGISGREGLQAARISDYSIAQFRFLQRLLFVHGRWNYLRTGKYILATFWKEIVFFLPQAHYQRFNGYTGTSLYENWSLTVFNTLFTSLPVIFLGAFEKDLSAETLLKYPELYSYGHRSSAINLWLYLGWTIMGIAESFVIYYITWGAYDKLPFDQDTSLYAMGTVPFTVCVVFINLRLLYVPIFFLHVFGDQWC.HGSRVLEMHTKTIISFLGVFLSVGGWFLWNLILSGIFEERLRIYQIRRAFIDNFGRTANFWAVVLVALGAVLVLELLVHAMRRVYFPHDVDLMQRVEREEKKALKRGVAVADAEVGDGAEALDAKDQGVMPRSADTSQQRLDVMQVEDGGDPFEKGFGKVKGAKKQQQKKKASRWLSKDSGGEETSIEMRATGGSRANV
